# Differentiation and fiber type-specific activity of a muscle creatine kinase intronic enhancer

**DOI:** 10.1186/2044-5040-1-25

**Published:** 2011-07-07

**Authors:** Phillip WL Tai, Katherine I Fisher-Aylor, Charis L Himeda, Catherine L Smith, Alexandra P MacKenzie, Deri L Helterline, John C Angello, Robert E Welikson, Barbara J Wold, Stephen D Hauschka

**Affiliations:** 1 Department of Biochemistry, 1705 NE Pacific St., University of Washington, Seattle, WA 98195, USA; 2 Division of Biology and Beckman Institute, 1200 E. California Blvd. California Institute of Technology, Pasadena, CA 91125, USA

## Abstract

**Background:**

Hundreds of genes, including muscle creatine kinase (*MCK*), are differentially expressed in fast- and slow-twitch muscle fibers, but the fiber type-specific regulatory mechanisms are not well understood.

**Results:**

Modulatory region 1 (MR1) is a 1-kb regulatory region within *MCK *intron 1 that is highly active in terminally differentiating skeletal myocytes *in vitro*. A *MCK *small intronic enhancer (*MCK*-SIE) containing a paired E-box/myocyte enhancer factor 2 (MEF2) regulatory motif resides within MR1. The SIE's transcriptional activity equals that of the extensively characterized 206-bp *MCK *5'-enhancer, but the *MCK*-SIE is flanked by regions that can repress its activity via the individual and combined effects of about 15 different but highly conserved 9- to 24-bp sequences. ChIP and ChIP-Seq analyses indicate that the SIE and the *MCK *5'-enhancer are occupied by MyoD, myogenin and MEF2. Many other E-boxes located within or immediately adjacent to intron 1 are not occupied by MyoD or myogenin. Transgenic analysis of a 6.5-kb *MCK *genomic fragment containing the 5'-enhancer and proximal promoter plus the 3.2-kb intron 1, with and without MR1, indicates that MR1 is critical for *MCK *expression in slow- and intermediate-twitch muscle fibers (types I and IIa, respectively), but is not required for expression in fast-twitch muscle fibers (types IIb and IId).

**Conclusions:**

In this study, we discovered that MR1 is critical for *MCK *expression in slow- and intermediate-twitch muscle fibers and that MR1's positive transcriptional activity depends on a paired E-box MEF2 site motif within a SIE. This is the first study to delineate the DNA controls for *MCK *expression in different skeletal muscle fiber types.

## Background

Muscle creatine kinase (*MCK*) is among the most abundant transcripts in striated muscle [1]. In differentiating muscle cell cultures, the onset of *MCK *expression occurs shortly after proliferating myoblasts exit the cell cycle [2] and begin to express differentiation-specific transcription factors [3]. In mouse embryos, *MCK *expression is initiated after the activation of myogenic transcription factors. *MCK *mRNA is first detectable in embryonic day 13 (E13) cardiac and skeletal muscles, and its expression is maintained throughout adulthood [4]. The expression of *MCK *between different anatomical muscle groups is quite variable; for example, MCK protein as well as its enzymatic product, creatine phosphate, are about two or three times higher in fast-twitch muscles than in slow-twitch muscles [5,6]. Fiber type-specific muscle regulatory factors (MRFs) have been studied in several other skeletal muscle genes, such as in *MLC2v*, *MLC1/3f *and *aldolase *genes [7-10] and even more extensively in *slow *and *fast troponin I *genes [11-16]. These studies have provided important clues that implicate a variety of transcriptional control mechanisms in muscle fiber type-specific gene expression. Aspects of these mechanisms are both similar to and different from those that regulate *MCK *expression in fast- and slow-twitch fiber types.

While *MCK *gene expression has been extensively studied [17-22], some of its regulatory regions have yet to be fully characterized. Currently, the 5'-enhancer (-1,256 to -1,050) is the best characterized of the known regions [18,20,23-28]. It has the ability (1) to drive high-level transcription of reporter genes in skeletal and cardiac muscle in both transgenic mice and cell culture and (2) to function with heterologous promoters [29]. Deletion and mutation analyses within this region in cultured skeletal myocytes and in transgenic mice have defined seven control elements: muscle-specific (CArG) and serum response element promoters, activator protein 2 (AP-2), Six4/5, AT-rich, left and right E-boxes and myocyte enhancer factor 2 (MEF2) [23,24]. The *MCK *proximal promoter (-358 to +1) has also been thoroughly studied. It is active in skeletal and cardiac myocytes in culture and can function independently of the 5'-enhancer. The proximal promoter is also active in transgenic skeletal muscle, and the combination of both the 5'-enhancer and the proximal promoter exhibits significant synergy in both cell culture and transgenic mice. The proximal promoter contains at least four active transcription factor binding sites: p53, E-box, CArG, and MPEX, a recently discovered sequence that recruits both Myc-associated zinc finger protein (MAZ) and Krupple-like factor 3 (KLF3) [30-33]

Studies involving the systemic delivery of expression constructs via adeno-associated vector type 6 vectors and transgenic mice have demonstrated that the *MCK *5'-enhancer and proximal promoter confer transcriptional activity several orders of magnitude higher in muscles containing primarily fast-twitch fibers, such as the tibialis anterior (TA) and quadriceps, than in muscles containing slow-twitch fibers, such as the diaphragm and soleus [22,34,35]. In contrast, the ratio of endogenous MCK protein levels in fast- to slow-twitch skeletal muscles is only about 2:1 [5,6,36]. The discrepancy between gene construct expression levels and endogenous MCK levels suggests that *MCK *gene transcription in slow-twitch fiber types is partially governed by regulatory elements located elsewhere in the *MCK *locus. This hypothesis is supported by previous transgenic tests of an approximately 6.5-kb mouse *MCK *gene region (-3,349 to +3,236) that was used to express dystrophin in *mdx *mice [37]. While fiber-type expression ratios were not included in these studies, the detection of dystrophin in all fibers implied that one or more subregions within the -3,349 to +3,236 sequence in addition to the 5'-enhancer and proximal promoter play major roles in *MCK *expression in slow- and intermediate-twitch muscle fibers.

The *MCK *gene locus also contains a less well-characterized 1-kb control region called modulatory region 1 (MR1), which resides within the +740 to +1,721 portion of the gene's first intron. In previous and very preliminary studies, MR1 was shown to promote muscle-specific transcription in skeletal myocyte cultures and in transgenic skeletal muscle [19,22,38]. We began the present study by comparing MR1 sequences among six mammalian species and discovered that MR1 is highly conserved throughout its sequence. Most of the conserved motifs are not sequences known to bind muscle gene transcription factors, but a 95-bp subregion within MR1, the *MCK *small intronic enhancer (*MCK*-SIE), was shown to contain conserved and functional E-box and MEF2 control elements, and chromatin immunoprecipitation (ChIP) assays and ChIP-Seq analyses demonstrate that the *MCK*-SIE's E-box and MEF2 elements interact with MyoD/myogenin and MEF2, respectively. The *MCK*-SIE exhibits much higher transcriptional activity than the entire MR1 in differentiated skeletal muscle cultures, and the SIE's elevated activity is due to removing it from the repressive effects of highly conserved regions flanking the *MCK*-SIE's 5'- and 3'-borders.

Upon discovering the enhancer-like properties of the *MCK*-SIE, and recalling that *MCK *transgenes containing only the 5'-enhancer and proximal promoter regions express relatively poorly in slow- and intermediate-twitch fibers, we hypothesized that expression of *MCK *in these fiber types may require the *MCK*-SIE-containing MR1 region. We therefore generated transgenic mouse lines that carry the 6.5-kb *MCK *regulatory region with or without MR1. Comparison of transgene fiber-type expression patterns between these lines supports our hypothesis. Interestingly, while E-box and MEF2 elements are common to other important regulatory regions in the *MCK*-SIE and the rat slow upstream regulatory element (SURE) region in *slow troponin I*, the key DNA control elements that ensure slow-twitch muscle fiber expression in the SURE region [11,13,14,39], are not present in the *MCK*-SIE (see Discussion).

## Results

### Sequence analysis of the intron 1 modulatory region MR1 reveals multiple highly conserved sequence motifs

**Figure 1:**
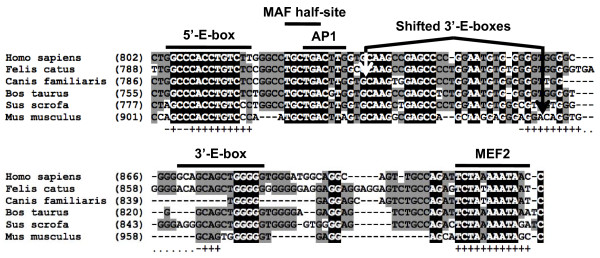
**Modulatory region 1 (MR1) contains a highly conserved subregion containing known myogenic control element motifs**. Sequence alignment of MR1 reveals a highly conserved 95-bp subregion, muscle creatine kinase (*MCK*) small intronic enhancer (*MCK*-SIE), that contains five putative control elements: an E-box motif pair, a myocyte enhancer factor 2 (MEF2) consensus motif and partially overlapping sequences that match proven MAF half-site and activator protein 1 (AP-1) sequences (see also Additional file 1 Figure S1). Bases that are identical in all six species (*Homo sapiens*, *Felis catus*, *Bos taurus*, *Sus scrota*, *Canis familiaris *and *Mus musculus*) are shown in black, while bases conserved between at least three species are shown in gray. The 3'-E-box is present in all six species, but is slightly more 5' in the mouse and further 5' in the dog. Conformation of mouse control element sequences to the MyoD/myogenin and MEF2 consensus sequences are indicated below the mouse sequence (+ = conforms, - = differs).

To begin our characterization of mouse MR1 and its role in *MCK *gene expression, a 1,081-bp region (+740 to +1,721) was aligned to the MR1 regions of five other mammalian species (human, cat, dog, bovine and pig) to reveal the presence of potentially functional control elements (Figure 1 and Additional file 1, Figure S1). This comparison revealed several MR1 subregions containing many highly conserved sequence motifs, which were then compared to a transcription factor binding motif library deposited in the TRANSFAC database [40]. Of particular interest was a 95-bp region (+901 to +995) that was subsequently proven to exhibit the properties of a transcriptional enhancer (Figure 1). The *MCK*-SIE exhibits high sequence conservation and contains four motifs known to control the transcription of many muscle genes: two core E-boxes (CAnnTG) [41,42], a MEF2 site and an overlapping MAF half-site and AP-1 site (Figure 1). Among six mammalian species, 11 to 12 bp of the more 5'-E-boxes conform to the 14-bp MyoD/myogenin consensus binding site: [C/G]N[A/G]_2 _CA[C/G]_2 _TG[C/T]_2 _N[C/G] [17] and 10 to 12 bp of the more 3'-E-boxes conform to the consensus binding sequence. Since the dog and mouse E-box sequences are located further 5' than in the other species (Figure 1), and since the distance between the 5'-E-box and MEF2 site varies from 16 to 40 bp, the precise distances between the four *MCK*-SIE control elements may not be functionally important. The MEF2 motif in all six species conforms fully to the MEF2 consensus sequence ([G/T][C/T]TA[A/T]_3 _ATA[A/G][A/C/T]) [43]. In addition, a region located near the 5'-E-box contains partially overlapping sequences that match perfectly with proven MAF and AP-1 binding sites [44]. The clustering of these motifs seems significant, since the combination of a paired E-box and MEF2/AT-rich motif has been observed in many muscle promoters, including the *MCK *5'-enhancer [45,46].

### MR1 is required for high-level *MCK *gene expression in differentiated skeletal muscle cells, and it contains a highly active SIE

**Figure 2:**
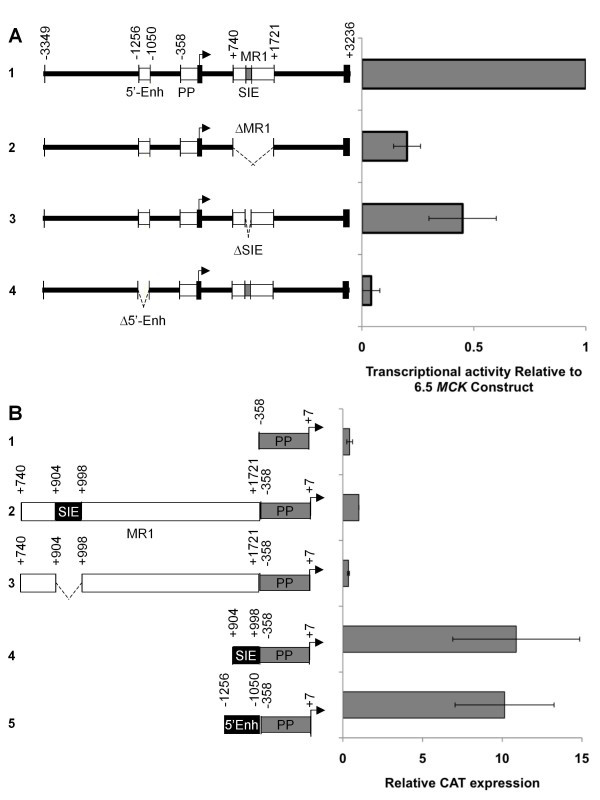
**MR1 is a positive regulator of *MCK *transcription**. (A) MM14 skeletal myocytes were cotransfected with an *MCK *enhancer-alkaline phosphatase (AP) reference plasmid and test gene plasmids containing the chloramphenicol acetyl transferase (CAT) reporter gene driven by the full-length 6.5-kb *MCK *construct (6.5*MCK-*CAT, #1), the 6.5-kb construct with MR1 deleted (6.5*MCK*ΔMR1-CAT, #2), the 6.5-kb construct with the *MCK*-SIE deleted (6.5*MCK*ΔSIE-CAT, #3) or, for comparison, the 6.5-kb construct with the 5'-enhancer deleted (6.5*MCK*ΔEnh-CAT, #4). Test construct activities are represented as the average values of relative CAT over AP activity normalized to the activity of 6.5*MCK-*CAT. (B) MR1 is composed of regions that promote transcription in MM14 cultures. Constructs containing the "full-length" MR1 (MR1-PP-CAT, #2), a construct lacking the *MCK*-SIE (MR1ΔSIE-PP-CAT, #3) or just the *MCK*-SIE (SIE-PP-CAT, #4) were generated to test the functional activity of the *MCK*-SIE. Activities of these test constructs were normalized to activities of the proximal promoter alone (PP-CAT, #1). The activity of the 5'-enhancer (5'Enh-PP-CAT, #5) is provided for comparison. Each experiment was performed in at least twelve plates in three separate experiments, and activities are averages of those experiments. Error bars represent ±1 standard deviation.

To address the function of MR1 in *MCK *gene expression, the MR1 region was deleted from the entire 6.5-kb *MCK *sequence (Figure 2A, constructs 1 and 2 [6.5*MCK*CAT and 6.5*MCK*ΔMR1-CAT]), and the effect of the deletion was examined in differentiated skeletal myocytes (MM14). To gauge the relative change in transcriptional activity caused by the loss of MR1, we compared 6.5*MCK*ΔMR1-CAT to a construct that contains a deletion of the well-characterized *MCK *5'-enhancer (Figure 2A, construct 4 [6.5*MCK*ΔEnh-CAT]). Expression from each test plasmid was normalized to the activity of a muscle-specific *MCK *enhancer-driven alkaline phosphatase (AP) reference construct.

Deletion of MR1 results in an approximately fivefold lower transcriptional activity in differentiated MM14 cultures than that produced by the entire 6.5-kb *MCK *gene construct (*P *< 0.01) (Figure 2A, constructs 1 and 2), whereas deletion of the *MCK *gene 5'-enhancer results in a greater than 10-fold decrease (*P *< 0.01).

To determine whether the *MCK*-SIE is critical for *MCK *gene transcription, it was deleted from the 6.5*MCK-*CAT construct and the resulting 6.5*MCK*ΔSIE-CAT was tested in differentiated skeletal muscle cultures (Figure 2A, construct 3). The deleted construct exhibited a 60% decrease in transcriptional activity in skeletal myocytes (*P *< 0.01), demonstrating that, in the context of the 6.5-kb *MCK *genomic sequence, the *MCK*-SIE is likely responsible for much of the positive transcriptional activity of MR1.

### *MCK*-SIE is active in differentiated skeletal muscle cells when placed 5' of the *MCK *proximal promoter

To facilitate further analysis of MR1 regulatory functions, subsequent studies were carried out in the context of MR1 placed 5' of the highly conserved *MCK *proximal promoter (Figure 2B (MR1-proximal promoter-chloramphenicol acetyl transferase (MR1-PP-CAT)), construct 2). This test construct frees MR1 from transcriptional effects of the highly active *MCK *5'-enhancer, which could lead to dampened effects of mutations or deletions within MR1. Importantly, it also avoids potential confounding effects due to cotranscriptional or posttranscriptional events, such as altered splicing efficiency or altered elongation efficiency, which could occur in conjunction with testing MR1 function within its 3' intron 1 location in the native *MCK *gene. In agreement with the decreased activity observed when MR1 is deleted from the 6.5-kb sequence (Figure 2A), MR1-PP-CAT exhibits transcriptional activity in skeletal myocyte cultures that is approximately threefold greater than that of the proximal promoter alone (Figure 2B, compare constructs 1 and 2). MR1's positive activity when moved 5' of the transcription start site also indicates that it has the properties of an enhancer.

Since the *MCK*-SIE had the greatest potential for explaining the positive activity of MR1 (Figure 2A), we tested its capacity to act as an enhancer independent of other MR1 sequences. Deletion of the *MCK*-SIE from MR1 reduces transcriptional activity to a level similar to that of the proximal promoter alone (Figure 2B, construct 3). Conversely, when the *MCK*-SIE was placed directly upstream of the proximal promoter (Figure 2B, M*CK*-SIE-PP-CAT, construct 4), a greater than 10-fold increase in transcription (*P *< 0.01) relative to the MR1-PP-CAT construct was observed. In fact, the *MCK*-SIE synergizes with the proximal promoter, as does the 5'-enhancer (Figure 2B, 5'Enh-PP-CAT, construct 5).

### Two E-box motifs and a MEF2 site are required for full transcriptional activity of the *MCK*-SIE in skeletal myocytes

**Figure 3:**
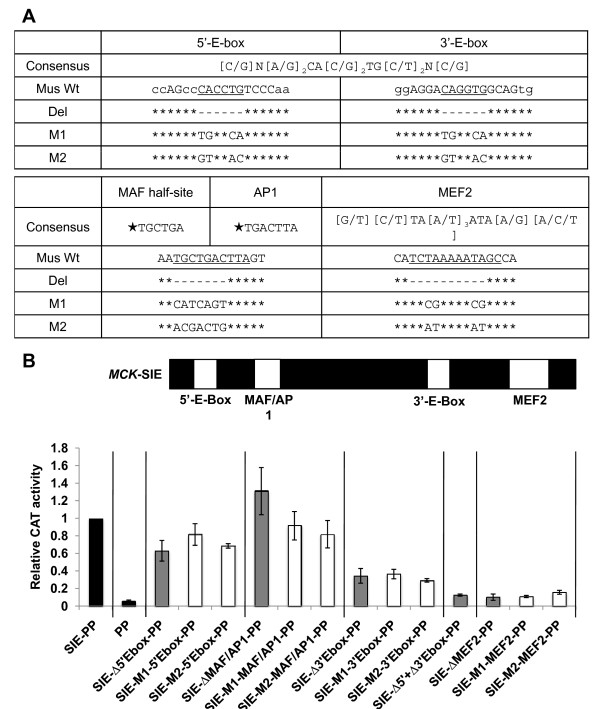
**Two E-boxes and a MEF2 site are critical for activity of the *MCK*-SIE**. (A) Deletions and mutations tested in *MCK*-SIE. The currently accepted consensus motifs for the E-box and MEF2 motifs are shown. Proven MAF half-site and AP-1 control element sequences are also indicated. Stars indicate sequences that were experimentally proven to recruit the labeled factors and do not represent consensus binding motifs. The wild-type mouse sequences of these elements within the *MCK*-SIE (Wt), the deletion sequences (Del) and two mutation sequences (M1 and M2) used in this study are shown on successive lines. Base pair deletions are indicated as hyphens, point mutations are shown as changed bases and asterisks indicate unchanged bases. (B) Mutational analysis of control elements within the *MCK*-SIE. The E-box, MAF/AP-1 and MEF2 motifs in the *MCK*-proximal promoter-CAT (*MCK*-SIE-PP-CAT) (diagrammed with elements in their relative positions) were deleted (gray bars) or subjected to two mutations (white bars) within core bases (Figure 2A) and were tested for transcriptional activity in differentiated MM14 skeletal myocyte cultures. The relative activities of these constructs were compared to the *MCK*-SIE-PP-CAT construct (scaled to equal 1.0) and PP-CAT alone (black bars). Each construct was tested in twelve plates in three separate experiments, and activities shown are averages of those experiments. Error bars represent ±1 standard deviation.

To determine the transcriptional activity of the *MCK*-SIE conserved binding site motifs, the 5'- and 3'-E-boxes and MEF2 motifs were subjected to both deletion and mutation analyses (Figure 3A) in the context of the *MCK*-SIE-PP-CAT construct (Figure 2B, construct 4). In skeletal myocytes, deletion or mutations of the 5'-E-box resulted in approximately 30% reductions in transcriptional activity, whereas deletion or mutations of the 3'-E-box resulted in approximately 65% reductions (Figure 3B), and deletion of both E-boxes caused a nearly 90% decrease in transcriptional activity. Deletion or mutations of the single MEF2 consensus motif also caused an approximately 90% reduction in transcriptional activity (Figure 3B). These data imply that both E-boxes contribute to the *MCK*-SIE's transcriptional activity, but that the 3'-E-box provides most of the activity. Since mutation of the MEF2 site leads to about the same loss in activity as mutation of both E-boxes, and since E-box binding factors are known to synergize with MEF2, it may be that the bulk of the *MCK*-SIE's transcription activity is derived from a single highly active MEF2-MyoD/myogenin complex.

The possibility that other control elements may reside in the *MCK*-SIE is raised by the highly conserved TGCTGAC[T/g]T[G/a]G sequence that begins several base pairs 3' of the 5'-E-box (Figure 1). The TGCTGA portion is a perfect match to MAF half-sites [47,48], and the TGACTTA sequence in the mouse *MCK*-SIE is a perfect match to a fully functional noncanonical AP-1 site [49,50]. Deletion and mutations that should have abolished the binding of either MAF or AP-1 (Figure 3A) had little to no effect on transcriptional activity (Figure 3B). This does not negate the possibility that MAF and/or AP-1 interactions within the *MCK*-SIE region play a role in *MCK *gene expression *in vivo*, but such interactions are not important for the *MCK*-SIE's transcriptional activity in differentiating skeletal myocyte cultures.

### Both MyoD and myogenin bind to the *MCK*-SIE in differentiated skeletal myocytes

On the basis of the rapid onset of *MCK *expression during differentiation, the transcriptional activity of MR1 in myocyte cultures (Figure 2B) and the presence of two active E-box elements within this region (Figure 3B), it seemed likely that MyoD and/or myogenin associate with the *MCK*-SIE. ChIP analysis of differentiating skeletal myocyte cultures was thus employed to determine whether the E-box pair recruits MyoD, myogenin or both MRFs *in vivo*.

One caveat of ChIP data interpretation is that control elements cannot be distinguished with respect to transcription factor binding when they bind the same factors and are close enough that both sites will be present on many of the same randomly sheared chromatin fragments. This would certainly be the case for the *MCK*-SIE E-box pair, where the separation is only 46 bp. Thus, primers that flank the entire *MCK*-SIE were used to detect MyoD- and myogenin-immunoprecipitated chromatin. This issue is also pertinent to ChIP discrimination between occupancy of the *MCK*-SIE E-box pair and other *MCK *E-boxes with proven transcriptional activity. These are centered at -1,175 and +1,152 within the *MCK *5'-enhancer and at -246 within the proximal promoter [26]. Therefore, in addition to using primers that amplify the *MCK*-SIE, primers for the 5'-enhancer were used as a positive control, since this region is known to contain two functional E-boxes that bind MyoD and myogenin [17,51,52].

**Figure 4:**
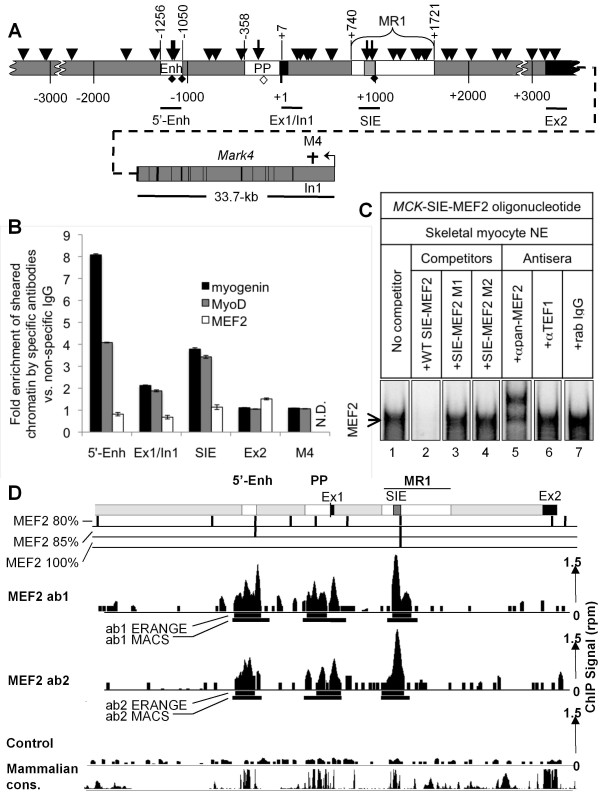
**MyoD and myogenin are enriched at the *MCK*-SIE in skeletal myocytes**. (A) Diagram of the 6.5-kb *MCK *regulatory region with the three known active regulatory regions: the 5'-enhancer, PP, MR1 (white boxes), the *MCK*-SIE (light gray box) exons 1 and 2 (black boxes) and other regions (gray), including the 33.7-kb *Mark4 *gene (located approximately 40 kb 3' of the *MCK*-SIE and transcribed in the opposite direction). E-box CAnnTG core motifs (arrowheads) occur throughout the 6.5-kb sequence. Among the thirty-five total E-boxes are two functional E-boxes within the 5'-enhancer, one functional E-box within the proximal promoter and two E-box motifs within the *MCK*-SIE (longer arrows). The less frequent MEF2 motifs (full diamonds) are found only in the 5'-enhancer and MCK-SIE and as a possible nonconsensus MEF2 site (open diamond) in the proximal promoter. The chromatin immunoprecipitation (ChIP) primer pairs (black lines) that span the 5'-enhancer sequence were used as positive controls for MyoD and myogenin binding to functional E-boxes. Negative controls consist of genomic regions containing either no core E-box motifs (region within the Mark4 intron 1 (M4, dagger)) or core E-box motifs with no proven transcriptional function (*MCK *gene exon 1/intron 1 boundary (two E-boxes) and exon 2 (one E-box); see Results, section-5). (B) MyoD and myogenin bind *MCK *gene E-box motifs. ChIP analyses using antibodies for MyoD, myogenin, MEF2 and control immunoglobulin G (IgG) were performed using chromatin from differentiated MM14 cell myocytes. The graph shows data from one of three ChIP experiments that is representative of the enrichment detected at each position by antibodies to myogenin (black bars), MyoD (gray bars) or MEF2 (white bars) over nonspecific rabbit IgG as determined by quantitative polymerase chain reaction (qPCR) assay. Error bars represent ±1 standard deviation of triplicate samples. (C) Electrophoretic mobility shift assay (EMSA) of MEF2 binding to the *MCK*-SIE MEF2 control element. Nuclear extracts from differentiated MM14 cultures were incubated with a ^32^P-labeled probe containing the *MCK*-SIE-MEF2 sequence with no competitor (lane 1), wild-type MEF2 competitor (lane 2), two different mutant MEF2 competitors (lanes 3 and 4), pan-MEF2 antibodies (lane 5), transcriptional enhancer factor 1 (TEF-1)-specific antibodies (lane 6) or nonspecific rabbit IgG (lane 7). Arrows indicate the MEF2-containing complex and free probe. (D) MEF2 ChIP-Seq occupancy at the 6.5-kb *MCK *regulatory region in differentiated C_2 _C_12 _cells shows that MEF2 is present at all three control regions. The 6.5-kb region is shown in schematic at the top (5'-enhancer, proximal promoter and MR1 are shown in white; *MCK*-SIE is shown in gray). Sequences that match the MEF2 canonical motif (CTAWWWWTAG) at the 80%, 85% and 100% thresholds are mapped throughout the 6.5-kb region. The sequenced and mapped ChIP signals (reads per million (rpm)) for the two pan-MEF2 antibodies 1 and 2 and the control (input DNA) are indicated as black histograms (scale shown at the right). Two different ChIP-Seq region finders (Model-based Analysis of ChIP-Seq data and Enhanced Read Analysis of Gene Expression) define the sequence range in which MEF2 is predicted to bind (see Materials and methods), and these are shown below each signal track as black bars. Conservation across the regions is shown from the University of California Santa Cruz (UCSC) Genome Browser plot of phastCons scores for the 20 default placental mammals.

Three "negative" control primers were used to rule out the possibility of cross-enrichment from factors binding to non-*MCK*-SIE regions (Figure 4A). The first "negative" control primer set amplifies intron 1 of the MAP/microtubule affinity-regulating kinase 4 (*Mark4*) gene. This sequence is roughly 40-kb 3' of the *MCK*-SIE on mouse chromosome 19 and is within a 1-kb region that entirely lacks the core E-box binding motif CAnnTG; thus it should serve as a truly negative control for MyoD and myogenin occupancy of the *MCK*-SIE. The second "negative" control primer pair spans the exon 1/intron 1 boundary and amplifies a 217-bp region located 690 bp upstream of the *MCK*-SIE, 242 bp downstream of the active promoter E-box and 1,149 bp downstream of the active *MCK *5'-enhancer right E-box (Figure 4A). The mouse exon 1/intron 1 boundary region contains two nonconserved E-boxes and also has four nonconserved E-boxes located 52, 67, 97 and 310 bp downstream of its 3'-border. None of these E-boxes have been tested for transcriptional activity, but they are likely to be transcriptionally inactive as they are not conserved in other mammals. Nevertheless, this would not preclude their occupancy by MyoD/myogenin or their function in mouse muscle cells; thus examining this subregion was also of interest in itself. The third "negative" control primer pair spans a 209-bp region starting at exon 2 (Figure 4A). It contains one nonconserved E-box and two other nonconserved E-boxes which are located 36 bp and 638 bp upstream of its 5'-border. MyoD/myogenin binding to any of these exon 2 E-boxes would thus cause an enrichment that would be detected by the exon 2 primer pair. Conversely, if MyoD and/or myogenin occupy the *MCK*-SIE, and if the negative control regions are not occupied, enrichments of the *MCK*-SIE and of the *MCK *5'-enhancer (positive control) should be significantly greater than those at any of the negative control regions.

Accordingly, ChIP analysis showed that antibodies for both MyoD and myogenin enriched the 5'-enhancer several-fold over nonspecific immunoglobulin G (IgG) (Figure 4B), and both antibodies also enriched the *MCK*-SIE region. In contrast, neither antibody enriched the exon 2 and *Mark4 *genomic regions significantly above nonspecific IgG. This demonstrates that MyoD and myogenin bind neither to nonconserved, and presumably nonfunctional, E-box motifs in the regions surrounding the *MCK*-SIE, nor to chromatin regions that lack E-boxes. There is a slight enrichment at the exon 1/intron 1 boundary. However, this could be caused by cross-enrichment due to MyoD and myogenin occupancy of the nearby and functional proximal promoter E-box [26], the 5'-enhancer, the *MCK*-SIE or any combination of these regions. Nevertheless, the enrichment due to MyoD and myogenin occupancy of the *MCK*-SIE region is probably not due to spurious enrichment from amplification of longer sheared chromatin fragments that include the 5'-enhancer or proximal promoter, because the enrichment signal from the exon 1/intron 1 region would then be higher than that of the *MCK*-SIE, and it is not. MyoD and myogenin thus occupy proven functional E-boxes in the 5'-enhancer and the *MCK*-SIE in differentiated skeletal myocytes, and they do not appear to occupy E-boxes in regions flanking the *MCK*-SIE. An additional consistent observation in these studies is that myogenin exhibits an approximately twofold higher occupancy of the 5'-enhancer than MyoD, whereas both MRFs exhibit equivalent occupancy of the *MCK*-SIE.

### MEF2 interaction with the *MCK*-SIE *in vitro *and *in vivo*

As demonstrated in Figure 3B, the MEF2 site contributes strongly to the transcriptional activity of the *MCK*-SIE region. Since members of the MEF2 superfamily of transcription factors (MEF2A, MEF2B, MEF2C and MEF2D) [53] have previously been shown to play important roles in muscle gene transcription, we asked whether any of the MEF2 family members were associated with the *MCK*-SIE *in vivo*. In initial ChIP analysis, several different MEF2 antibodies unexpectedly failed to enrich the *MCK*-SIE or even the 5'-enhancer (Figure 4B) (see Discussion). Furthermore, antibodies to octamer binding protein 1 (Oct-1) and transcriptional enhancer factor 1 (TEF-1), two factors known to transactivate AT-rich motifs in muscle promoters [54,55] and known to be present in myocyte cultures, also failed to precipitate the *MCK*-SIE when used in ChIP assays (data not shown). This led us to question whether MEF2 in our cell culture model was detectable by immunoassays.

To establish that differentiated MM14 cultures contain MEF2 protein, that MEF2 protein is recognized by the pan-MEF2 antibody used in our ChIP study and that MEF2 can indeed bind to the *MCK*-SIE, we analyzed MEF2 binding by electrophoretic mobility shift assay (EMSA). ^32^P-labeled *MCK*-SIE-MEF2 sequence probes were generated and incubated with MM14 nuclear extracts. Gel electrophoresis with the *MCK*-SIE-MEF2 probe revealed a single intense band, which implied that either a single or multiple factors of similar size were bound to the *MCK*-SIE-MEF2 probe (Figure 4C). Wild-type competitor oligonucleotides completely abolished this band, whereas two oligonucleotides containing different mutations of the *MCK*-SIE-MEF2 motif had no effect. Furthermore, a partial supershift of the band was caused when the probe was incubated with nuclear extracts in the presence of a pan-MEF2 antibody, suggesting that the band of interest contains MEF2. The partial shift likely occurred because the entire complex might not be fully and stably accessible by the antibody to produce a consistent supershift. These results argue that MEF2 proteins are present in the nuclei of differentiated MM14 muscle cells, that MEF2 is capable of binding to the *MCK*-SIE probe and that MEF2 antibodies, which did not precipitate *MCK*-SIE-enriched sequences in ChIP analysis, were capable of binding MEF2 oligonucleotide complexes in EMSA studies of similarly differentiated muscle cultures.

Since TEF-1 also binds AT-rich motifs in muscle gene promoters and has been shown to bind the *MCK *5'-enhancer [55], we asked whether TEF-1 binds to the MEF2 sequence in the *MCK*-SIE. Incubation with TEF-1-specific antisera did not supershift or abolish the "MEF2 complex," whereas it did supershift a TEF-1-specific complex (data not shown). A nonspecific IgG also failed to alter the mobility or intensity of the MEF2-specific band (Figure 4C). The absence of detectable MEF2 binding in our ChIP study (Figure 4) is therefore not likely to be due to competitive *in vivo *occupancy of the MEF2 site by TEF-1.

As MEF2 occupancy of the *MCK *5'-enhancer has been reported in mouse embryos and in the B22 myogenic cell line following Brahma-related gene 1 and MyoD induction [42], it seemed possible that unknown differences between the myogenic states of the different cell culture models might affect the ability to detect MEF2 occupancy in the *MCK *locus. Fortunately, ChIP-Seq analyses aimed toward identifying genome-wide MEF2 binding events in terminally differentiated muscle cells were being performed in parallel studies by the Wold group (personal communication, B. Wold). We therefore collaborated in analyzing the *MCK *locus. Initial ChIP-Seq experiments in C_2 _C_12 _skeletal muscle cells also failed to detect significant MEF2 ChIP signals at the *MCK *locus or at several other MEF2 target loci, thus suggesting that MEF2 might be inefficiently cross-linked to DNA under standard ChIP conditions. Since members of the MADS family of transcription factors, of which MEF2 is a member, often depend significantly on protein-protein interactions with other DNA-bound factors, and since the MyoD family of factors interact with MEF2 through protein-protein interactions [56], we reasoned that chromatin fixation conditions designed to more efficiently stabilize these interactions might improve ChIP detection (see Materials and methods).

Following the modified fixation procedure, a standard sequencing readout from this material revealed distinct MEF2 signals at the *MCK*-SIE and at the 5'-enhancer (Figure 4D). These signals were very similar in biological replicate chromatin samples that had been immuno-enriched by MEF2 antibodies directed against nonoverlapping epitopes (data not shown). Enrichment over background was more than 10-fold at both sites (P < 2e-13 for Model-based Analysis of ChIP-Seq data (MACS) and P < 8e-7 for Enhanced Read Analysis of Gene Expression (ERANGE)), and no other site in the *MCK *locus was significantly occupied, except for the dispersed signals observed throughout the *MCK *proximal promoter sequence. Enrichment of MEF2 within the proximal promoter, which contains no sequences that match the canonical motif (although one with 80% similarity is present (Figure 4D)), could be due to any of several possibilities (see Discussion). The observed MEF2 ChIP-Seq peaks overlap regions of high-sequence conservation among placental mammals at the 5'-enhancer, the proximal promoter and the *MCK*-SIE regions as determined by phastCons scores, which predict evolutionarily conserved elements using a 30-species vertebrate sequence alignment and phylogenetic tree information (Figure 4D).

### MR1 contributes to *MCK *gene expression in slow- and intermediate-twitch fiber types in adult mice

**Figure 5:**
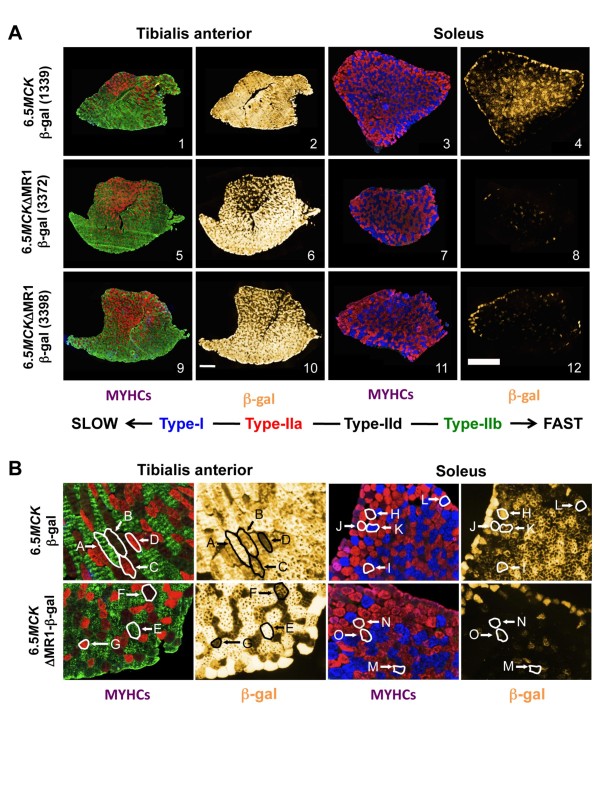
**MR1 is important for *MCK *expression in slow- and intermediate-twitch skeletal muscle fibers**. (A) Sister sections of tibialis anterior (TA) and soleus muscles from mice carrying the 6.5*MCK*-β-gal or the 6.5ΔMR1-β-gal transgenes, immunostained with myosin heavy chain (MYHC) fiber type-specific monoclonal antibodies (panels 1, 3, 5, 7, 9 and 11) or activity stained for β-galactosidase (β-gal) expression (panels 2, 4, 6, 8, 10 and 12). Antibodies for different isoforms and fluorophore-labeled secondary antibodies mark the fiber types as follows: slow-twitch fibers (type I), blue; intermediate-twitch fibers (type IIa), red; and fast-twitch fibers (types IIb and IId), green and black, respectively (the black appearance of type IId fibers is due to the absence of any type 1, IIa, or IIb antibody binding). Purplish fibers contain both types I and IIa MYHCs (see Figure 5B, soleus), and fibers with weak red or green staining probably contain mixtures of type IId (no color) + type IIa or type IId + type IIb, respectively (see Figure 5B, TA). Sister sections were stained for β-gal expression (false colored gold). Bars are 0.5 mm. (B) Higher magnification sections indicate differences in β-gal expression between fiber types in transgenic lines with and without MR1. Individual fibers, outlined in white or black to show relative differences in X-gal staining between fiber types (type I = K, L and O; type IIa = C, D, G, I and J; type IId = B, F, H and M; and type IIb = A and E), can be cross-referenced to β-gal expression in sister sections.

Previous investigations of *MCK *gene regulation in transgenic mice have suggested that the 5'-enhancer and the proximal promoter are highly active in anatomical muscles with predominantly fast-twitch fibers (type IIb and type IId (also called type IIx or type IId/x fibers)) such as the TA muscle. Conversely, the activity levels of the 5'-enhancer and the proximal promoter were at least 10-fold lower in muscles from the same transgenic mice that contained a high proportion of slow-twitch muscle fibers (type I) or intermediate-twitch muscle fibers (type IIa) such as soleus [26,27]. Since the endogenous levels of MCK protein in fast vs. slow muscle fibers differ by only about threefold [5], the previous transgenic studies implied that regulatory regions in addition to the 5'-enhancer and proximal promoter are required for full *MCK *expression in slow-twitch fibers. This led us to hypothesize that MR1 may contribute to *MCK *expression in type I and type IIa fiber types. To test this possibility, we generated transgenic mouse lines containing either the 6.5-kb *MCK *genomic region driving the β-galactosidase (β-gal) reporter gene (6.5*MCK-*β-gal) or the same construct lacking MR1 (6.5*MCK*ΔMR1-β-gal). Adult transgenic mice were killed, and TA and soleus muscles were dissected and cryosectioned. Sections were then X-gal-stained to detect β-gal transgene expression. To identify the specific fiber types expressing β-gal, we adopted a method of visualizing the four distinct fiber types on a single sample section by immunofluorescence tagging of myosin heavy chain (MYHC) isotypes as described by Gregorevic *et al*. [57] (see Discussion for rationale of MYHC vs. histochemical fiber typing). Sister sections were thus immunostained with monoclonal antibodies that recognize the MYHC isoforms found in slow-twitch muscle fibers (type I), intermediate-twitch muscle fibers (type IIa) and fast-twitch muscle fibers (type IIb) (Figures 5A and 5B). Type IId fibers were identified based on the absence of immunostaining with all of the above-mentioned monoclonal antibodies [58]. It should be noted that the distribution of fiber twitch types assessed by MYHC isotype expression within the anatomical muscles examined among different transgenic lines was qualitatively similar (data not shown). Thus introduction of the transgenes themselves did not alter the distribution of fiber twitch types. Whether expression levels of the wild-type 6.5*MCK*-β-gal and 6.5*MCK*DMR1-β-gal transgenes are differentially affected by the metabolic states within individual muscle fiber types remains to be determined.

Comparisons between immunostained and X-gal-stained sister cross-sections of the TA and soleus muscles of mice carrying the 6.5*MCK-*β-gal transgene showed β-gal expression in all fiber types, but there was a clear difference in the distribution of X-gal staining intensities among fiber types in the predominantly fast-twitch TA muscles compared to the predominantly slow- and intermediate-twitch soleus muscles (Figure 5A, panels 2 and 4). As a general rule in TA muscle, type IIb fibers exhibit greater X-gal staining than type IId fibers, and type IIa fibers exhibit the least staining (Figure 5B, TA X-gal panel, fiber staining intensities: A > B > C), whereas in the soleus, type IId and type IIa fibers exhibit the greatest X-gal staining and type I fibers stain the least (Figure 5B, soleus X-gal panel, fiber staining intensities: H > I > K).

Interestingly, fibers that show similar MYHC expression can also vary in X-gal staining intensity (compare TA fibers C with D and soleus fibers I with J and K with L). However, the overall trend found within the same transgenic mouse and even within the same anatomical muscles is that the 6.5*MCK-*β-gal transgene is more active in individual fast-twitch muscle fibers than in intermediate- and slow-twitch fibers. These β-gal/fiber-type staining patterns were consistent among all mice tested (*n *= 7) in the single 6.5*MCK-*β-gal-transgenic line.

Four transgenic mouse lines that contain the 6.5-kb regulatory region lacking MR1 (6.5*MCK*ΔMR1-β-gal) exhibit a strikingly different β-gal expression profile. In the TA, there is weaker relative X-gal staining in regions of the TA that are dominated by type IIa fibers (Figure 5A; compare panels 5, 6, 9 and 10 with panels 1 and 2). At higher magnification, this difference can be directly correlated with low levels of X-gal staining in type IIa fibers (Figure 5B, TA panels, fiber G and others) and reduced staining in some type IId fibers (Figure 5B, TA panels, fiber F and others). However, in the same TA muscle, type IIb fibers (Figure 5, fiber E and others) stain intensely for β-gal. In the soleus muscle, X-gal staining is relatively weak throughout the section in comparison to similarly treated TA muscle sections (Figure 5A, panels 7, 8, 11 and 12 vs. panels 3 and 4). At higher magnification, both type I and type IIa muscle fibers show very weak X-gal staining (Figure 5B, soleus panels, fibers N, O and others), while the few fibers that express β-gal are type IId fibers (Figure 5B, soleus panels, fiber M and others). These observations were consistent among all mice tested (*n *= 7) from the four independent 6.5*MCK*ΔMR1-β-gal-transgenic lines. This suggests that MR1 contributes strongly to the expression of *MCK *in type I and type IIa fibers, and perhaps weakly in type IId fibers, but that MR1 is not absolutely required for high-level *MCK *expression in type IIb fibers.

Expression levels from the wild-type 6.5*MCK-*β-gal and 6.5*MCK*ΔMR1-β-gal transgenes were also examined in protein extracts from entire anatomical muscles containing different proportions of fast and slow fibers. Extensor digitorum longus (EDL) muscles (primarily fast-twitch fibers) and soleus muscles (primarily slow-twitch and intermediate-twitch fibers) were dissected from four or five mice each from the most highly active lines carrying each transgene, and β-gal specific activity was determined. In all mice from each transgenic line, EDL extract activities were significantly higher than those from the soleus. However, because absolute expression levels typically differ between individual transgenic mouse lines, owing to variable transgene integration sites and copy numbers [25-27], the β-gal-specific activity levels were then normalized for each line by dividing the EDL levels by the soleus levels. The ratio was three times higher in extracts from the 6.5*MCK*ΔMR1-β-gal-transgenic mice (data not shown). In combination with the much lower transgene expression levels observed within the individual type I and type IIa fibers of 6.5*MCK*ΔMR1-β-gal-transgenic mice (Figure 5), the quantitative data are consistent with the conclusion that the MR1 region plays a relatively more important role in *MCK *gene expression in muscles containing slow and intermediate fiber types than in muscles containing primarily fast fibers.

## Discussion

In this study, we characterized the *MCK *intronic region MR1 [22] and found that it contains regulatory elements that provide positive transcriptional activity in skeletal muscle cells. Our results argue that MR1 is crucial for the "full" activity of the 6.5-kb *MCK *regulatory region in differentiated skeletal muscle cultures (Figure 2), and they recapitulate those of an earlier study that demonstrated MR1's ability to drive transcriptional activity in a position-independent manner [22]. Additionally, we found that MR1's positive transcriptional activity is conveyed by a highly conserved 95-bp sequence designated the *MCK*-SIE (Figure 1). When separated from its flanking MR1 regions, the *MCK*-SIE synergizes with the proximal promoter to provide transcriptional activity equivalent to that of the highly active *MCK *5'-enhancer (Figure 2B) [22]. Interestingly, however, the *MCK*-SIE requires the 358-bp *MCK *proximal promoter for its activity, whereas the 5'-enhancer exhibits high activity with the 80-bp *MCK *basal promoter as well as with the proximal promoter (data not shown).

The *MCK*-SIE's high activity is largely due to the paired E-box and MEF2 motifs, since their mutation or deletion caused a significant decrease in transcription, while mutations affecting the AP-1/MAF half-site motifs did not (Figure 3). Although a TRANSFAC database search of the mouse *MCK *gene's 1-kb MR1 region revealed many possible transcription factor binding motifs, and although many of these overlap with conserved sequences (Additional file 1, Figure S1), deletion of other conserved regions did not disclose a correlation with positive transcriptional activity (Additional file 1, Figure S1, and Additional file 2, Figure S2). While it is also possible that some aspects of MR1-mediated *MCK *expression are regulated by nonconserved control elements, as we have shown is the case for Six4/5 and *MAZ *elements in the 5'-enhancer and proximal promoter [24,32] and as has been shown for other genes [59,60], pursuing this possibility did not seem as immediately fruitful as investigating the SIE's E-box and MEF2 mechanisms. Nevertheless, our studies do not preclude positive transcriptional contributions from other MR1 and SIE sequences.

Several ChIP studies have indicated the ability of E-box motifs in skeletal muscle gene promoters to recruit the basic helix-loop-helix factors MyoD and myogenin, and EMSA studies have proven E-box binding by Myf5, MRF4 and E12/47 as well [45]. Analysis of early phases of muscle differentiation also suggests that MyoD may bind muscle gene promoters as a "pioneering" factor [3] that facilitates histone acetylation [45]. As differentiation progresses, MyoD is then replaced by myogenin at the same regulatory regions. This was shown to be the case for the *MCK *5'-enhancer in E10.5 to E14.5 mouse limb muscles [51]. This transition may be facilitated by decreased levels of Suv39h1, a histone H3 lysine 9-specific methyltransferase that represses myogenin expression via histone and MyoD methylation [61]. However, in our ChIP studies of MM14 muscle cultures harvested four days after the initiation of differentiation, a time at which 90% of the myonuclei are in MYHC-positive cells, both MyoD and myogenin were detected at the 5'-enhancer as well as at the *MCK*-SIE (Figure 4B). These data demonstrate that a rapid and complete MyoD-to-myogenin binding transition is not observed in the cell culture system used in our study. However, it may be informative that we found the ratio of myogenin to MyoD enrichment of the 5'-enhancer to be consistently greater than that of the *MCK*-SIE, where about equal ChIP signals were detected. The biological relevance of this difference in enrichment is not yet understood.

Our *MCK*-SIE ChIP data for differentiating MM14 cultures are generally consistent with ChIP-Seq studies that have probed the entire genomic occupancy of MyoD in differentiated mouse C_2 _C_12 _myocytes [52] in that both studies detected enriched MyoD occupancy of the *MCK*-SIE, proximal promoter and 5'-enhancer. Our data are also consistent with a temporal ChIP-Seq data set showing no MyoD or myogenin occupancy of the *MCK*-SIE in replicating C_2 _C_12 _cells and highly enriched occupancy by both factors in fully differentiated cultures (A. Kirilusha, G. Kwan and B. Wold, personal communication). On the basis of our mutagenesis studies, the *MCK*-SIE 3'-E-box appears to be the more active site, since its deletion caused a greater reduction of transcriptional activity (Figure 3B). This might be attributed to the mouse 3'-E-box's being a closer match (12 of 14 bp) to the overall E-box consensus sequence than the 5'-E-box (11 of 14 bp) (Figure 1C). Alternatively, the closer proximity of the 3'-E-box than the 5'-E-box to the MEF2 site may improve the synergistic interactions between MyoD/myogenin and MEF2 and may lead to greater activity of the 3'-E-box In either case, it is not known whether one or both E-boxes preferentially associate with MyoD or myogenin *in vivo *or whether this might change under different physiological conditions. Ideally, this question could be addressed by ChIP analysis, but because the two E-boxes are only 46 bp apart, their individual occupancies cannot be definitively resolved on the basis of currently available data. Our *MCK *locus-specific MyoD/myogenin ChIP data also concur with the global ChIP-Seq MyoD data [52] with respect to occupied and unoccupied E-boxes in the sense that the strongly preferred sequence for occupied E-boxes in differentiated C_2 _C_12 _muscle cultures is CAG/cCTG. All of the occupied E-boxes in our study conformed to this sequence, and no unoccupied E-boxes within the *MCK *regions studied had the preferred sequence. It is also worth emphasizing that even though dozens of CAnnTG consensus E-boxes occurred within the 6.5-kb *MCK *genomic region, and while some of these occurred in clusters of two or three E-boxes within a 100-bp region (Figure 4A), neither our study nor the more comprehensive global ChIP-Seq study (personal communication, B. Wold). detected significant MyoD binding at the vast majority of these E-boxes. This indicates that the mere presence of one or more nearby E-box motifs within transcriptionally active muscle gene promoters does not imply their functionality. Conversely, since our laboratory has proven the function of E-boxes within all three of the *MCK *genomic regions in which ChIP and ChIP-Seq detected significant MyoD binding, the data suggest that the detection of reproducible MyoD ChIP peaks of this type in muscle genes is strongly indicative of transcriptional function of the associated E-boxes. While the ChIP studies implicate MyoD and myogenin as binding to the *MCK*-SIE and 5'-enhancer E-boxes, it is important to point out that cell culture studies are not necessarily indicative of the MRFs that occupy these E-boxes in adult skeletal muscle fibers. In the latter context, it is likely that these E-boxes may be primarily occupied by MRF4, since it appears to be the predominant MRF in adult skeletal muscle [62,63].

The *MCK*-SIE MEF2 site is also critical for transcriptional activity, as removing this sequence is even more deleterious than removing the individual E-boxes (Figure 3B). Consistent with this, we found that MEF2 binds this sequence *in vitro *by EMSA using nuclear extract from MM14 myocytes (Figure 4C). Furthermore, ChIP-Seq studies of differentiated C_2 _C_12 _muscle cells identified enriched MEF2 occupancy at both the 5'-enhancer and the *MCK*-SIE (Figure 4D), and the fold enrichments at these sites relative to the negative control were more than 10-fold. A diffuse signal over the proximal promoter region was also observed, and this signal may reflect either that binding to a nonconsensus MEF2 site or that MEF2 association with MyoD/myogenin bound to a proximal promoter E-box located at -247 bp provides positive transcriptional activity both *in vitro *and *in vivo *[25,27]. Alternatively, MEF2 enrichment at the proximal promoter may be due to the secondary binding of MEF2 complexes formed at the 5'-enhancer and/or the *MCK*-SIE physically contacting the promoter. Such long-distance interactions of enhancer-affiliated factors with promoter DNA via cross-linking with initiation complex proteins have been readily detected in standard ChIP reactions during chromatin conformation capture [64].

Overall, we conclude that MEF2 interacts *in vivo *with the *MCK*-SIE complex. The strong dependency of *MCK*-SIE function on the presence of the MEF2 control element (Figure 3B) also supports the hypothesis that MEF2 likely binds directly at this site. The functional synergy of this MEF2 site with E-box control elements bound by MyoD and myogenin is reminiscent of the behavior of an analogous E-box pair and MEF2 site in the *MCK *5'-enhancer [23] and is consistent with a model of cobinding involving MEF2 and MRFs [46,56,65], although simultaneous occupancy by both factors *in vivo *is inferred and has not been directly measured.

Interestingly, all four isoforms of MEF2 (MEF2A, MEF2B, MEF2C and MEF2D) are present in myocyte cultures [53], but MEF2B is not present in adult mouse muscle [66,67]. The *MCK*-SIE sequence does not predict which, if any, MEF2 isoforms bind preferentially [53], and the antibodies used in our ChIP assays cross-reacted with all MEF2 isoforms. Thus, it is possible that the MEF2 site may be occupied by any of the MEF2 isoforms present in differentiated skeletal muscle cultures. It is also plausible that the MEF2 site can be occupied by other non-MEF2 factors that recognize AT-rich motifs. For example, AT-rich motifs similar to the one found in the *MCK*-SIE are known to bind nuclear factors such as Oct-1, TEF-1 and MHox [24,51,55,68-72], and the *MCK *5'-enhancer's MEF2 and AT-rich motifs have been shown to recruit MEF2, Oct-1 and TEF-1. In this regard, even though the *MCK *5'-enhancer and *MCK*-SIE contain similar paired E-box/MEF2 motifs, the *MCK*-SIE fails to bind TEF-1 by EMSA analysis (Figure 4C), whereas the 5'-enhancer MEF2 element binds TEF-1 [55]. Although the functional consequences of this difference are unknown, these data imply that the MEF2 site-mediated transcriptional activity of the *MCK*-SIE and *MCK *5'-enhancer may differ in terms of their interactions with non-MEF2 factors.

An intriguing facet of MR1's regulatory function is the discovery that it contains transcriptionally repressive sequences flanking the highly positive *MCK*-SIE. These MR1 regions can repress the *MCK*-SIE's activity via the combined or individual effects of at least 15 highly conserved 9- to 24-bp sequences (Figure 2B and Additional file 1, Figure S1, and Additional file 2, Figure S2). When MR1 constructs containing individual deletions of these motifs were tested in skeletal muscle cultures, most of the deletions resulted in two- to fourfold increases in transcriptional activity (Additional file 2, Figure S2), suggesting that these conserved regions act to repress transcriptional activity. The only deletion that resulted in a significant decrease in activity overlapped the MEF2/AT-rich motif within the *MCK*-SIE region (Additional file 1, Figure S1, and Additional file 2, Figure S2). Interestingly, deletion F, which encompassed the *MCK*-SIE's conserved 5'-E-box, did not cause decreased activity when tested in the context of the entire MR1 region (Additional file 2, Figure S2), but did lead to decreased activity in the context of the isolated *MCK*-SIE (Figure 3B). This may be due to the compensatory functions of other control elements within the entire MR1.

Our studies have also begun to address the *in vivo *function of MR1 in *MCK *gene expression. Comparisons between a transgenic mouse line that contains the 6.5-kb sequence driving β-gal and several lines from which the MR1 region has been deleted revealed differences in transgene expression that indicated a correlation between MR1 function and muscle fiber type. Transgenic lines expressing the 6.5*MCK*ΔMR1-β-gal transgene expressed very low levels of β-gal in slow- and intermediate-twitch fibers (type I and type IIa), while expression levels in fast-twitch fibers (type IIb and type IId) remained high (Figure 5). Although only one wild-type 6.5*MCK-*β-gal-transgenic line was derived in our own study, an independent transgenic study that employed the same 6.5-kb *MCK *genomic sequence to express the transcriptional enhancer factor domain family member 1 (TEAD1) transcription factor demonstrated high-level transgene expression in the soleus (slow- and intermediate-twitch muscle fibers) as well as in EDL (fast-twitch muscle fibers) [73].

Our transgenic analysis of *MCK *gene regulation has focused on correlations between transgene expression levels and fiber types defined according to their MYHC isotype expression profiles. Since *MCK *functions in an energy transport pathway that is important for optimal contractile function, it might also have been informative to identify fiber types based on metabolic markers such as succinate dehydrogenase and nicotinamide adenine dinucleotide phosphate levels that could be detected via histochemical assays and then to correlate these fiber types with transgene expression levels. This was not done for purely technical reasons, as MYHC immunostaining provided more precise distinctions between fiber types and because the ability to detect four fiber types in a single cryosection facilitated correlations between fiber types and β-gal levels in adjacent sections. Furthermore, since the original investigators of muscle fiber types based on MYHC immunostaining were very careful to ascertain that individual fibers were designated as the same fiber type by both the histochemical and immunostaining protocols [58], it seems likely that our study would have reached similar conclusions regarding the role of MR1 in *MCK *gene expression with either fiber-typing technique.

There is clearly a functional relationship between *Myhc *types and *MCK *gene expression patterns [6,74], but the underlying basis of this regulatory linkage is not known. In this regard, however, the distribution of MYHC isotypes in different anatomical muscle is not altered in *MCK*-deficient mice; rather, the lack of *MCK *appears to be compensated by an increase in mitochondrial creatine kinase (CK) [75]. Recently, it has also been shown that the expression patterns of myosin isoforms and enzymes involved in muscle fiber energy metabolism can be uncoupled by mutations that affect glycogen storage and sarcoplasmic calcium release mechanisms [76]. These reports suggest that *MCK *transgene expression would not be anticipated to exhibit a strict correlation with muscle fiber types as assessed solely by MYHC fiber typing. This possibility may partially explain why the *MCK*-driven β-gal levels observed in transgenic TA and soleus muscles were not uniform among all fibers of each MYHC-defined type (Figure 5B). These nonuniformities in transgene expression within specific fiber types do not appear to be regulated by MR1, since they are observed in fibers carrying the intact 6.5-kb *MCK *genomic region as well as in those in mice carrying the 6.5*MCK*ΔMR1 transgene. Nevertheless, the MR1 region clearly plays an important role in regulating the steady-state levels of *MCK *gene expression in different anatomical muscles and in different fiber types. In this regard, it has yet to be determined whether MR1 or the *MCK*-SIE alone can drive expression in slow- and intermediate-twitch muscle fibers independently of the 5'-enhancer. It is also not known which physiological signals impinge on the *MCK*-SIE and on the flanking repressive regions within MR1.

Transgenic analysis of fiber type-specific muscle gene expression has also been carried out with the *MLC2v*, *MLC1/3f*, *aldolase A *and *slow troponin I *muscle genes [7-10,14]. Similarly to our studies with *MCK*, E-boxes and MEF2 control elements have been identified within their key regulatory regions. In particular, the *slow troponin I *SURE region contains the critical E-box, MEF2, and a CACC motifs, which in isolation confer pan-muscle expression. Interestingly, the inclusion of a more upstream region within SURE, which contains a *bicoid*-like motif that recruits the general transcription factor 3 (GTF3)/muscle transcription factor II I repeat domain-containing protein 1 (MusTRD1), restricts activity to slow-twitch muscle [11,14]. A related mechanism may modulate the *in vivo *activity of the *MCK*-SIE, leading to the contribution of MR1 to expression in slow-twitch fibers. However, neither the *bicoid*-like motif (GTTAATCCG) [14] nor the GTF3 consensus DNA binding sequence (G_TC _G_A _GATTA_G _BG_A _) [11] is found in or immediately adjacent to the *MCK*-SIE. In contrast, the fast-twitch activity of the *MCK *5'-enhancer may be partially due to recruitment of the Six4 transcription factor, since the MEF3 site in the *aldolase A *pM promoter is necessary but not sufficient to drive transcription in some fast-twitch muscle fibers [77].

The contribution of multiple enhancer regions to the expression of striated muscle genes in different fiber types may be a common mechanism. For example, transgenic analysis has demonstrated that the troponin I (fast) enhancer intronic regulatory element (TnIfast IRE), in isolation, results in fast twitch fiber-specific expression in the adult plantaris muscle, where TnIfast IRE elements yield the highest levels of expression in type IIb fibers, intermediate levels in type IId, very low levels in type IIa fibers and no expression in type I fibers [16], while the endogenous *TnIfast *gene is expressed at similar levels in all fast-twitch fiber types [15]. The *MCK *gene MR1 region, although its activity contributes to expression in slow and intermediate fibers, appears analogous to TnIfast IRE in that both regulatory regions provide relatively restricted fiber-type expression patterns and both genes require the contribution of multiple fiber-specific enhancers to achieve pan-skeletal muscle expression. The *MCK *MR1 and 5'-enhancer regulatory regions thus appear to share common mechanisms of transcription with several fast- and slow-twitch muscle genes.

## Conclusions

This study identifies a regulatory region within the *MCK *gene's intron 1 that plays a major transcriptional role in slow- and intermediate-twitch muscle fibers. This activity was shown *in vitro *to be dependent on the *MCK*-SIE region, which contains a paired E-box and MEF2 motif. Each motif was shown to be required for full *MCK*-SIE transcriptional activity, and ChIP studies showed that they recruit MyoD, myogenin and MEF2, respectively. It was also shown that the *MCK*-SIE is flanked by repressive regulatory regions containing multiple different negative control elements. The mechanisms and functional purposes of these remain to be determined.

## Materials and methods

### Sequence analysis

Sequences spanning the TATA box to exon 2 of the *MCK *gene of *Homo sapiens *(human [AC005781.1]), *Felis catus *(cat [GenBank: AC135221.3AC135221.3]), *Canis familiaris *(dog [GenBank: AC137538.2]), *Bos taurus *(bovine [GenBank: AC137535.2]), *Sus scrofa *(pig [GenBank: AC139878.2]) and *Mus musculus *(mouse GenBank: [AC118017.15]) were obtained from compiled genomic sequences in the Entrez Genome Project database and subjected to sequence alignment using ClustalW [78]. The intron 1 sequences of both mouse and human were independently analyzed for putative control element motifs using Match http://www.gene-regulation.com/cgi-bin/pub/programs/match/bin/match.cgi) (Contact B. Wold for specifics: http://woldb@caltech.edu, a matrix search algorithm that scours the TRANSFAC database of transcription factors and their experimentally proven binding sites. Parameters were set to select for vertebrate-only matrices with a 90% core binding similarity to broaden the rate of positive hits.

### Plasmid constructs

A 6.5-kb construct of the mouse *MCK *gene (-3,349 to +3,230) [37] was cloned upstream of the CAT reporter gene 6.5*MCK-*CAT [26]. The 6.5*MCK*ΔMR1-CAT construct was generated from the 6.5*MCK-*CAT construct by introducing *Cla*I restriction sites 5' and 3' of MR1 (+740 and +1,724) using the QuikChange Site-Directed Mutagenesis Kit (Stratagene, http://www.genomics.agilent.com/), according to the manufacturer's directions. MR1 was then deleted by digestion of the plasmid with *Cla*I and religation of the remaining vector. The 6.5*MCK*ΔEnh-CAT construct was generated by site-directed mutagenesis to delete the *MCK *5'-enhancer (-1,256 to -1,040).

The MR1 region was polymerase chain reaction-amplified from the existing 6.5-kb construct with primers containing the restriction sites *Sph*I (5') and *Bst*I (3'). The MR1 amplicon was cloned upstream of the proximal promoter by replacing the 5'-enhancer in the e-358-CAT reporter construct [27] using *Sph*I and *Bst*I. The mouse *MCK *PP region used in these studies extends from -358 to +7. All other deletions and mutations described in this study were generated using the QuikChange Site-Directed Mutagenesis Kit.

### Transient transfections and reporter gene assays

MM14 skeletal myoblasts were cultured as described previously [79]. Collagen-coated 100-mm dishes were inoculated with about 1 × 10^5 ^log phase cells/dish and were allowed to proliferate under growth conditions (85% Ham's F10C nutrients + gentamicin, 15% horse serum and 2 ng/mL basic fibroblast growth factor (bFGF) added at approximately 12-hour intervals) for about 24 hours. Myoblasts were cotransfected using a standard calcium phosphate method [23] at about 3 × 10^5 ^cells with test constructs driving the expression of the CAT reporter gene and an AP reference plasmid, which contains the 5'-enhancer placed 5' of the basal promoter sequence (-80 to +7). Transfected MM14 cultures were induced to differentiate four hours after beginning the transfection by aspirating the growth medium, rinsing once with saline G, incubating for 2 minutes at room temperature in 15% glycerol 4-(2-hydroxyethyl)-1-piperazineethanesulfonic acid-buffered saline, rinsing again with saline G and then adding 10 mL of differentiation medium (98.5% Ham's F10C nutrients + gentamicin, 1.5% horse serum and 1 μM insulin) [79]. Relative enzymatic activities of CAT and AP were determined from extracts as described in previous studies [25]. Since the *MCK *enhancer-AP reference plasmid is expressed only in differentiated muscle cells, it provides a control for plate-to-plate variability in transfection efficiency and extent of muscle differentiation in skeletal myocyte cultures.

### ChIP assays

ChIP assays were performed using a modification of the Fast-ChIP method as described previously [32,80] with the following nuances: 100-mm dishes were plated with about 1 × 10^5 ^log phase MM14 cells/dish and grown to near confluence (about 4 × 10^6 ^cells/dish), then allowed to differentiate in proliferation medium without additional bFGF for four to six days prior to harvesting. All cultures contained more than 90% terminally differentiated myocytes as assessed by immunostaining a parallel culture with the myosin-specific antibody MF-20. This procedure produced more than 7 × 10^6 ^differentiated myonuclei per 100-mm dish. Cells were sonicated with 16 rounds of 15-second pulses with 45 seconds of rest between pulses (four minutes total) on a Model 100 Sonic Dismembrator (Fisher Scientific, http://www.fishersci.com/) at the highest setting. Antibodies used for immunoprecipitation described in this study were as follows: anti-myogenin (M-225) sc-576 X, anti-MyoD (M-318) sc-760 X, anti-MEF2A (C-21) sc-313 and normal rabbit IgG sc-2027 (Santa Cruz Biotechnology, http://www.scbt.com/). The primers used in ChIP analyses were *MCK *5'-enhancer: 183 bp forward: 5'-GCCCATGTAAGGAGGCAAGGCC-3', reverse: 5'-CACCAGGGACAGGGTTATTTTTAGAGC-3', *MCK *exon 1/intron 1 boundary: 217 bp forward: 5'-GGGTCACCACCACCTCCACAG-3', reverse: 5'-GCCTTGCAAGGAGGGGACACTTG-3', *MCK*-SIE: 168 bp, forward: 5'-CTTGAGGCCCAGAGCCTGGCTG-3', reverse: 5'-GAGACCCAAAGCCCTTGAAGCTGCTAC-3', *MCK *exon 2: 207 bp, forward: 5'-GTCCCAAAGGCCGCCACCATG-3', reverse: 5'-GGGTTGTCCACCCCAGTCTGG-3' *Mark4 *gene region: 205 bp, forward: 5'-GGATGCCATGCCTGGTGGCCAT-3', reverse: 5'-GCCATGCAGCTTTCACGCAGAGG-3'.

### EMSA

EMSA was carried out as previously described [32]. Nuclear extracts from differentiated skeletal muscle cultures were prepared as previously described [81] using a cocktail of several protease inhibitors (P8340; Sigma, St. Louis, MO, USA). Total protein in the extracts was quantitated by using the Bradford method [82]. Incubations with antisera or unlabeled oligonucleotide competitors were carried out at room temperature for 20 minutes prior to the addition of probe. The 5' to 3' sequences of the double-stranded probes or competitors with introduced mutations of the sequence underlined are MEF2 (*MCK*-SIE): AGGAGCATCTAAAAATAGCCACAAAG, MEF2 (*MCK*-SIE)-M1: AGGAGCATCCGAAAACGGCCACAAAG and MEF2 (*MCK*-SIE)-M2: AGGAGCATCATAAAAATGCCACAAAG.

Antibodies used for EMSA were anti-MEF2A (pan-MEF2, C-21) (Santa Cruz Biotechnology), anti-TEF-1 (BD Transduction Laboratories, http://www.bdbiosciences.com/home.jsp) and IgG normal rabbit sc-2027 (Santa Cruz Biotechnology, http://www.scbt.com/).

### ChIP-Seq assays

ChIP assays for MEF2 ChIP-Seq were performed according to the protocol described by Johnson *et al*. [83] with the modifications described in the paragraph below. C_2 _C_12 _cells were grown at low density on Nunclon 14-cm-diameter plates (Fisher Scientific, http://www.fishersci.com/) in 20% fetal bovine serum (FBS)/Dulbecco's modified Eagle's medium (DMEM) (#11965; Invitrogen http://www.invitrogen.com/site/us/en/home.html with penicillin and streptomycin and passaged at no more than 50% confluence. Upon reaching confluence, differentiation was induced by switching to 2% horse serum/1 μM insulin/DMEM. After 60 hours of differentiation, the cells were cross-linked with 1% formaldehyde (Avantor Performance Materials, http://www.avantormaterials.com/) and 0.025% glutaraldehyde (Polysciences, Inc. http://www.polysciences.com/) for 10 minutes. A total of 2 × 10^7 ^cells were fragmented to about 100 to 300 bp using 30-second, 12-W cycles on a Misonix 3000 sonicator http://www.fishersci.com/ecomm/servlet/fsproductdetail?aid = 2819374&storeId = 10652 for a total sonication time of 15 minutes. The sheared chromatin was immunoprecipitated using 100 μL of sheep anti-mouse IgG M280 beads (Invitrogen) and 5 μg of MEF2 monoclonal antibody (clone B4) from Santa Cruz Biotechnology or 200 μL of sheep anti-rabbit IgG M280 beads and 10 μg of MEF2 polyclonal antibody (clone H300) from Santa Cruz Biotechnology. Illumina libraries for sequencing were made using their ChIP-Seq library kit (Illumina, Inc., http://www.illumina.com/) as described by the manufacturer, except that a 10-cycle amplification was performed before gel selection according to the method of Johnson *et al*. (library 2) [83]. Library sequencing was performed for 36 cycles on an Illumina Genome Analyzer (Illumina, Inc.), and the resulting reads were mapped to the mouse MM9 genome by using Bowtie software [84]. Mapped reads that permitted up to two mismatches to the reference genome were displayed on the University of California Santa Cruz (UCSC) Genome Browser. ChIP-Seq signals were called using the ChIP-Seq module within the ERANGE version 3.2 software package [85] and were also mapped using the MACS peak caller [86].

### Transgenic mice

The 6.5-kb *MCK *gene sequence and the sequence with MR1 deleted were cloned upstream of the β-gal reporter gene to generate the 6.5*MCK-*β-gal and 6.5*MCK*ΔMR1-β-gal constructs, respectively. DNA for microinjection was prepared by enzymatic digestion to linearize the plasmids and gel-purified by freeze-and-squeeze columns (Bio-Rad Laboratories, http://www.bio-rad.com/). Transgenic mice were produced using eggs from C57BL/6J × C3H crosses through the University of Washington Transgenic Resource Program. Founders were crossed to C57BL/6J to generate F1s. Lines of mice analyzed were either F1s or the progeny of F1s (N2 or N3) that were back-crossed with C57B/6J.

### Dissections

Adult mice (1+ months) were killed according to methods approved by the University of Washington Institutional Animal Care and Use Committee. TA and soleus muscles were dissected and mounted in a 2:1 mixture of optimal cutting temperature compound and 10% gum tragacanth in cryomold cassettes. Cassettes were then frozen in liquid nitrogen-cooled isopentane. Tissues contained in blocks were cryosectioned at a thickness of 6 μM at -25°C using a Leica cryostat http://www.leica-microsystems.com/, mounted onto glass slides at room temperature and then stored at -80°C.

### X-gal staining

Slides were fixed in 4% paraformaldehyde in phosphate-buffered saline (PBS) for 15 minutes at 4°C and washed in 100 mM sodium phosphate (pH 7.3), 2 mM MgCl_2 _, 0.01% sodium oxycholate and 0.02% Nonidet P-40 and stained in a standard X-gal reagent solution [87] for 4 hours. After staining, slides were fixed for 15 minutes in 10% formalin and mounted in gelvatol (Sigma-Aldrich, http://www.sigmaaldrich.com). Images were obtained using a Zeiss Axiovert 200 microscope http://www.zeiss.com/micro with a Zeiss AxioCam MRm camera (Zeiss), and acquired using AxioVision software (Zeiss). Images were then uniformly false-colored using Adobe PhotoShop version 7 software (Adobe Systems, http://www.adobe.com/).

### Immunofluoresence

Monoclonal antibodies specific for myosin isoforms MYHC1, MYHC2A and MYHC2B were produced from cultures of hybridoma lines BA-D5, SC-71 and BF-F3, respectively [58]. These antibodies stain type I, type IIa and type IIb fibers, respectively. Cultures were grown to high density in DMEM High Glucose (HyClone Laboratories, http://www.hyclone.com/) supplemented with 10% FBS (Gemini Bioproducts, http://www.gembio.com/)) and penicillin-streptomycin (Sigma). Cultures were then switched to serum-free medium and incubated for two or three days. The medium was collected, centrifuged and filter-sterilized (0.22 μm Stericup; Millipore, http://www.millipore.com/) and monoclonal antibodies were concentrated by HiTrap column chromatography (GE Healthcare Biosciences, http://www.gelifesciences.com/). High-protein concentration fractions as determined by the Bradford method [82] were pooled and dialyzed (Slide-A-Lyzer Dialysis Cassettes; Pierce Biotechnology, http://www.piercenet.com/), and then stored at -20°C. Slides were treated with blocking buffer (1% bovine serum albumin and 0.05% Tween 20 in PBS) and incubated with about 10 μg/mL BA-D5, SC-71 and BF-F3 for 1 hour, washed three times for five minutes in blocking buffer and incubated with goat anti-mouse secondary antibodies IgG2b Alexa Fluor 350, IgG1 Alexa Fluor 594 and IgM Alexa Fluor 488 (Invitrogen) for 30 minutes. Slides were washed as before, rinsed in PBS and mounted in gelvatol. Images were acquired as described above.

## Abbreviations

AP: alkaline phosphatase; bFGF: basic fibroblast growth factor; β-gal: β-galactosidase; *BRG1*: Brahma-related gene 1; CAT: chloramphenicol acetyl transferase; ChIP: chromatin immunoprecipitation; ERANGE: Enhanced Read Analysis of Gene Expression; KLF3: Krupple-like factor 3; *Mark4*: MAP/microtubule affinity-regulating kinase 4 gene; MAZ: Myc-associated zinc finger protein; MEF2: myocyte enhancer factor 2; *MCK *and MCK: muscle creatine kinase gene and protein; MCK, *MCK*-SIE: *MCK *small intronic enhancer; MR1: modulatory region 1; MYHC: myosin heavy chain; Oct-1: octamer-binding protein; TA: tibialis anterior muscle.

## Competing interests

The authors declare that they have no competing interests.

## Authors' contributions

PWLT carried out the sequence alignments; made the test gene constructs; carried out the transfection assays, the ChIP analyses, the immunohistochemistry and immunofluorescence assays; and drafted parts of the manuscript describing Hauschka Lab data. KIFA conceived of the redesign of the ChIP-Seq fixation, performed and analyzed MEF2 ChIP-Seq and drafted portions of the manuscript describing Wold Lab data. CLH carried out the EMSA study and helped to draft the manuscript. CLS participated in the immunohistochemistry and immunofluorescence assays. APM participated in the transfection analyses. DLH carried out the whole muscle extract transgene expression assays. JCA and REW prepared and labeled the MYHC monoclonal antibodies and participated in the immunohistochemistry assays. BJW conceived of the global ChIP-Seq analysis of multiple myogenic transcription factors, participated in the design and coordination of the MEF2 ChIP-Seq studies and drafted parts of the manuscript describing the Wold Lab data. Together with PWLT, SDH conceived of the overall study, participated in its design and coordination between the two laboratories and played a major role in writing the manuscript. All authors read and approved the final manuscript.

## Supplementary Material

Additional file 1 **Figure S1. A six-species sequence alignment of modulatory region 1 (MR1), which demonstrates the conserved nineteen subregions throughout the region**. The MR1 sequences of six mammalian species (human, cat, dog, bovine, pig and mouse) were aligned to reveal sequence conservation. Bases that are fully conserved between the six species are highlighted in black, while those conserved in three to five species are highlighted in gray. Gaps in the sequence alignment are represented as hyphens. The 5' and 3' flanks of MR1, as defined in this study, are marked with red right-angled arrows. Nineteen conserved subregions (A-S, annotated by orange barbed lines) were tested for transcriptional activity (see Additional file 2, Figure S2). The two E-box elements, the MAF/activator protein 1 (AP-1) site and the myocyte enhancer factor 2 (MEF2) consensus sequence investigated in this study are outlined in green. The 1,081-bp MR1 region (+740 to +1,721) extends slightly more 5' and 3' than the originally described mouse MR1 sequence (+748 to -1,607) [29]. Click here for file

Additional file 2 **Figure S2. The functional consequence of individual deletions of the conserved 19 subregions throughout MR1**. (A) Conserved regions within MR1 (gray blocks in part A, gray bars in part B) were deleted from MR1-proximal promoter-chloramphenicol acetyl transferase (MR1-PP-CAT) and tested for transcriptional activity in skeletal myocytes (gray bars). (B) MM14 cells were transiently transfected with constructs containing each of the 19 different conserved motif deletions, and cells were harvested as described in the Figure 2 legend. Relative CAT activity was normalized with the *MCK *5'-enhancer alkaline phosphatase (AP) reference plasmid and compared to the intact MR1-PP-CAT (black bar) and to the PP-CAT (white bar). Expression levels of MR1-PP-CAT were scaled to equal 1.0. Asterisks indicate constructs that did not result in a statistically significant change in transcriptional activity. Click here for file
