# Supplementary material for: Differentiation and fiber type-specific activity of a muscle creatine kinase intronic enhancer
Source: Skelet Muscle. 2011 Jul 7;1:25. doi: 10.1186/2044-5040-1-25 (PMC3157005; doi:10.1186/2044-5040-1-25)
Supplement: Additional file 1 — Figure S1. A six-species sequence alignment of modulatory region 1 (MR1), which demonstrates the conserved nineteen subregions throughout the region. The MR1 sequences of six mammalian species (human, cat, dog, bovine, pig and mouse) were aligned to reveal sequence conservation. Bases that are fully conserved between the six species are highlighted in black, while those conserved in three to five species are highlighted in gray. Gaps in the sequence alignment are represented as hyphens. The 5' and 3' flanks of MR1, as defined in this study, are marked with red right-angled arrows. Nineteen conserved subregions (A-S, annotated by orange barbed lines) were tested for transcriptional activity (see Additional file 2, Figure S2). The two E-box elements, the MAF/activator protein 1 (AP-1) site and the myocyte enhancer factor 2 (MEF2) consensus sequence investigated in this study are outlined in green. The 1,081-bp MR1 region (+740 to +1,721) extends slightly more 5' and 3' than the originally described mouse MR1 sequence (+748 to -1,607) [29]. [file 2044-5040-1-25-S1.PDF]

## Figure S1

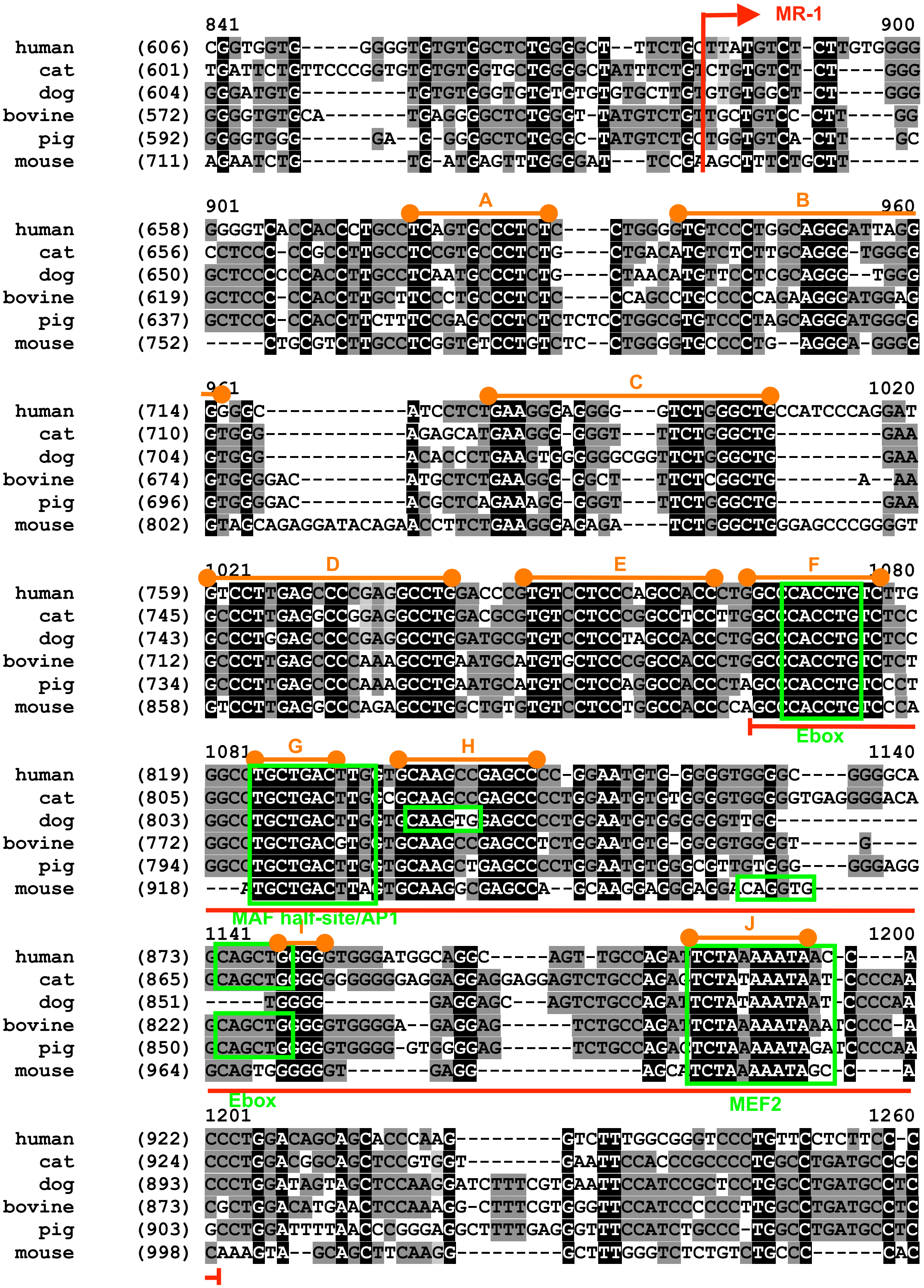
MR-1
841 900
human (606) CGGTGGTG-----GGGGTGTGTGGCTCTGGGGCT--TTCTGCTTATGTCT-CTTGTGGGG
cat (601) TGATTCTGTTCCCGGTGTGTGTGGTGCTGGGGCTATTTCTGTCTGTGTCT-CT----GGG
dog (604) GGGATGTG---------TGTGTGGGTGTGTGTGTGTGCTTGTGTGTGGCT-CT----GGG
bovine (572) GGGGTGTGCA-------TGAGGGGCTCTGGGT-TATGTCTGTTGCTGTCC-CTT----GG
pig (592) GGGGTGGG------GA--G-GGGGCTCTGGGC-TATGTCTGCTGGTGTCA-CTT----GC
mouse (711) AGAATCTG---------TG-ATGAGTTTGGGGAT---TCCGAAGCTTTCTGCTT------
901 A B 960
human (658) GGGGTCACCACCCTGCCTCAGTGCCCTCTC----CTGGGGTGTCCCTGGCAGGGATTAGG
cat (656) CCTCCC-CCGCCTTGCCTCCGTGCCCTCTG----CTGACATGTCTCTTGCAGGG-TGGGG
dog (650) GCTCCCCCCACCTTGCCTCAATGCCCTCTG----CTAACATGTTCCTCGCAGGG--TGGG
bovine (619) GCTCCC-CCACCTTGCTTCCCTGCCCTCTC----CCAGCCTGCCCCCAGAAGGGATGGAG
pig (637) GCTCCC-CCACCTTCTTTCCGAGCCCTCTCTCTCCTGGCGTGTCCCTAGCAGGGATGGGG
mouse (752) -----CTGCGTCTTGCCTCGGTGTCCTGTCTC--CTGGGGTGCCCCTG--AGGGA-GGGG
961 C 1020
human (714) GGGGC------------ATCCTCTGAAGGGAGGGG---GTCTGGGCTGCCATCCCAGGAT
cat (710) GTGGG------------AGAGCATGAAGGG-GGGT---TTCTGGGCTG---------GAA
dog (704) GTGGG------------ACACCCTGAAGTGGGGGGCGGTTCTGGGCTG---------GAA
bovine (674) GTGGGGAC---------ATGCTCTGAAGGG-GGCT---TTCTCGGCTG-------A--AA
pig (696) GTGGGGAC---------ACGCTCAGAAAGG-GGGT---TTCTGGGCTG---------GAA
mouse (802) GTAGCAGAGGATACAGAACCTTCTGAAGGGAGAGA----TCTGGGCTGGGAGCCCGGGGT
1021 D E F 1080
human (759) GTCCTTGAGCCCCGAGGCCTGGACCCGTGTCCTCCCAGCCACCCTGGCCCACCTGTCTTG
cat (745) GCCCTTGAGGCCGGAGGCCTGGACGCGTGTCCTCCCGGCCTCCTTGGCCCACCTGTCTCC
dog (743) GCCCTGGAGCCCCGAGGCCTGGATGCGTGTCCTCCTAGCCACCCTGGCCCACCTGTCTCC
bovine (712) GCCCTTGAGCCCCAAAGCCTGAATGCATGTGCTCCCGGCCACCCTGGCCCACCTGTCTCT
pig (734) GCCCTTGAGCCCCAAAGCCTGAATGCATGTCCTCCAGGCCACCCTAGCCCACCTGTCCCT
mouse (858) GTCCTTGAGGCCCAGAGCCTGGCTGTGTGTCCTCCTGGCCACCCCAGCCCACCTGTCCCA
Ebox
1081 G H 1140
human (819) GGCCTGCTGACTTGGTGCAAGCCGAGCCCC-GGAATGTG-GGGGTGGGGC----GGGGCA
cat (805) GGCCTGCTGACTTGCCGCAAGCCGAGCCCCTGGAATGTGTGGGGTGGGGGTGAGGGGACA
dog (803) GGCCTGCTGACTTGGTGCAAGTGGAGCCCCTGGAATGTGGGGGGTTGG------------
bovine (772) GGCCTGCTGACGTGGTGCAAGCCGAGCCTCTGGAATGTG-GGGGTGGGT-----G----
pig (794) GGCCTGCTGACTTGGTGCAAGCTGAGCCCCTGGAATGTGGGCGTTGTGGG----GGGAGG
mouse (918) ---ATGCTGACTTAGTGCAAGGCGAGCCA--GCAAGGAGGGAGGACAGGTG---------
MAF half-site/AP1
1141 I J 1200
human (873) GCAGCTGGGGGTGGGATGGCAGGC-----AGT-TGCCAGATTCTAAAATAAC-C----A
cat (865) GCAGCTGGGGGGGGGGAGGAGGAGGAGGAGTCTGCCAGAGTCTATAAATAAT-CCCCAA
dog (851) -----TGGGG---------GAGGAGC---AGTCTGCCAGATTCTATAAATAAT-CCCCAA
bovine (822) GCAGCTGGGGGTGGGGA--GAGGAG------TCTGCCAGATTCTAAAATAAATCCCC-A
pig (850) GCAGCTGGGGGTGGGG-GTGGGGAG------TCTGCCAGACTCTAAAATAGATCCCCAA
mouse (964) GCAGTGGGGGGT-------GAGG--------------AGCATCTAAAATAGC-C----A
Ebox
MEF2
1201 1260
human (922) CCCTGGACAGCAGCACCCAAG---------GTCTTTGGCGGGTCCCTGTTCCTCTTCC-C
cat (924) CCCTGGACGGCAGCTCCGTGGT--------GAATTCCACCCGCCCCTGGCCTGATGCCGC
dog (893) CCCTGGATAGTAGCTCCAAGGATCTTTCGTGAATTCCATCCGCTCCTGGCCTGATGCCTC
bovine (873) CGCTGGACATGAACTCCAAAGG-CTTTCGTGGGTTCCATCCCCCCTTGGCCTGATGCCTC
pig (903) GCCTGGATTTTAACCCGGGAGGCTTTGAGGGTTTCCATCTGCCC-TGGCCTGATGCCTC
mouse (998) CAAAGTA--GCAGCTTCAAGG---------GCTTTGGGTCTCTGTCTGCCC------CAC

```
                     K                 L
             1261                                                        1320
human    (972) TGGCTTCAGCTCACCG-TGGTCCCAATCTCCCTCCCACAATCCCACCTGCTTCCCATCCC
cat      (976) AGCCTTCGGCTCCCCCCTGGTCCTGATTCCCTTCCCACG-TCCTACCTGCCTCCCA----
dog      (953) AGCCTTCAGCTCGCC--TGGTCCCGATTCCCTTCCCACCGTCCTACCTGCTTCCCATC--
bovine   (932) GGCCTTCAGCTCACCC-TCGTGCCCATCTCCTTCCCACCGTCCTGCCCACTTCCCAT---
pig      (962) AGCCTTCAGCTCACCT-TGGTCTGCATCCCCTTCCTACCATCCTGCCTGCGTCCCAT---
mouse   (1041) ACTCTTCT-CTCAGCT-TGGTCCACCTTCCC-TCTCACCTTCCT--CTGAGGCCCC----

                   M
             1321                                                        1380
human   (1031) CGTCTCCCACCGCTGCTGGAGGCCCCTTCTC-----------------CTTTCTGG--GTC
cat     (1031) ---CTCCTGGAGC--CGGGAG---CGAGCTC-----------------CCA-------GTC
dog     (1009) -----CCTGGGGCCGC-GGAG---TGAGCTC-----------------CCA-------CGC
bovine   (988) ----TCCTGGAGCT--GGGAAATGAGCATCTCGTCCCTACCACCACCGCCAAAGGGTCCTC
pig     (1018) ----TCCCGGAGCT---GGAAAT-GAGTGTC-----------------CCA-------CTC
mouse   (1092) ---CTTCCAGCCCCGATGGAGGCCTGATGTCCCCCATGGTCAGTGCTTCAGGGATCTAGT

             1381                                                        1440
human   (1073) CCTTCTCCTTTCTGGGTCCAGTGAA--TAAAATAATAATAACAGTAGGACCTGG------
cat     (1060) CCGCCAC--------GGCCACTGAA--GA--------------GTCCCTCCTC-------
dog     (1037) CGACCACC------------------------------------ACCACCTC-------
bovine  (1042) CCTCCTCCTGGGTCTTGGGAATAAAAGTAAGAACAGCACAGCAGCCACCCCTCTAGGTGT
pig     (1047) CCACTGC---------------T-AA--------------GGG-GTCCCTTCTCC------
mouse   (1149) CAATAAAATTAATAATGAAAAACAACAGTAATAAAATACACGTGACGTGACTGGGGCAGC

             1441                                                        1500
human   (1125) ------------------CGCAGTGG-----CTCAC---ACGCATAATCCCAGCATCTTG
cat     (1089) ------------------CTTCCTGG-----CTCTT---ACGGATAAAC-----------
dog     (1053) ------------------CTTCCTGG-----GTC--------------------------
bovine  (1102) GCGCGCTAGTTTCAGACTCTTTGTGG-----CACTATGGACTGTTAGCCCACCAGGCTCT
pig     (1071) --------------------TTCTGG-----GTCTTGTGAATAAAAGT------------
mouse   (1209) TTAGGGCTTAGTTCAAATCCCAGTGTTCACACCCTTTAAAAGACAAGACAAAACAAAACA

             1501                                                        1560
human   (1159) GGAGGCCGATG----------------CTGGAGGATCACTTGAGGCCAGGAGT-------
cat     (1112) ----------G----------------CTAAACGC---------GACAGTA---------
dog     (1064) -----------------------------------------------CAGTA---------
bovine  (1157) TCTGTCCAAGGGAT------------TCTCCAGGCAAGAATACTGGAAGGGGTTGTCATT
pig     (1094) -----------A---------------------------------ACAGTAA--------
mouse   (1269) GCTGGCTGTGGGGGAGAACATCAGAATCCCCCTGGGGAGGTGGGGACAGGGGATCTGTGG

             1561                                                        1620
human   (1196) ---------CCCAGACCAGCCTGGGCAACATAGTGAGACCCCATCTCT-GCAAAAAAGTA
cat     (1128) ---------ACCACAGCGGCCCG---------------------CCCT-CCA--------
dog     (1069) ------------ACGGCAGCCAC--------------------CCCCT-AC---------
bovine  (1205) TCCTCCTCTAGGAGATCTTCCTGATCCAAGGATTGAGCTCTCATCTCTTACATCTCCTGC
pig     (1102) -----C--AATAATGGCAGCCATG-------------------CCTCG------------
mouse   (1329) GGCTCCATGGCCAG-CCAGCCTAGCTCCAGGCCTGCGAGAGA--CCCTACCTCAAGA---

             1621                                                        1680
human   (1246) GTAATAACAAATAACAATAATAGCAGCCACCAGGGTTCCAGGCTC-CAACTCC--ATGCC
cat     (1149) ----------------------------------GGTAGCAGGCCC-CAGCTCC--ACGGG
dog     (1087) -----------------------------------GGAGCAGGCCC-CAACTCC--ATGTG
bovine  (1265) ATTGGCA------G--------GCA---GGTTCTTTACAAGGCCC-CAGCTCC--ATGCC
pig     (1124) ---------------------------A-----GGTACGTG-----CAGTTCC--ACGCT
mouse   (1383) -TAAAATAAAATAAAATAAAATAAATATATAAAATAACAATCTTGCAGCACCTGAGGTC
```

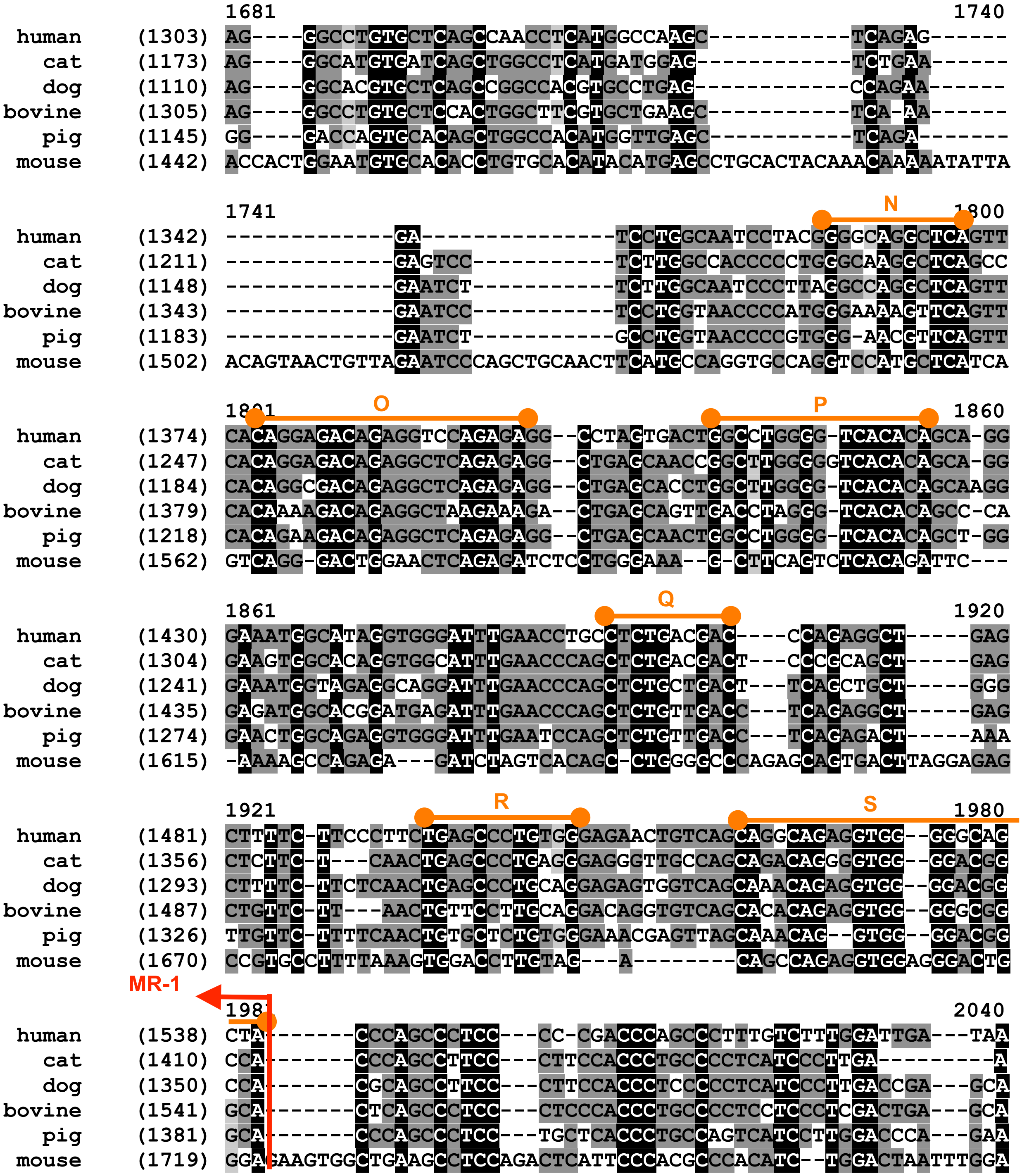

1681 1740
human (1303) AG----GGCCTGTGCTCAGCCAACCTCATGGCCAAGC------------TCAGAG------
cat (1173) AG----GGCATGTGATCAGCTGGCCTCATGATGGAG-------------TCTGAA------
dog (1110) AG----GGCACGTGCTCAGCCGGCCACGTGCCTGAG-------------CCAGAA------
bovine (1305) AG----GGCCTGTGCTCCACTGGCTTCGTGCTGAAGC------------TCA-AA------
pig (1145) GG----GACCAGTGCACAGCTGGCCACATGGTTGAGC------------TCAGA-------
mouse (1442) ACCACTGGAATGTGCACACCTGTGCACATACATGAGCCTGCACTACAAACAAAAATATTA
N
1741 1800
human (1342) --------------GA----------------TCCTGGCAATCCTACGGGGCAGGCTCAGTT
cat (1211) --------------GAGTCC------------TCTTGGCCACCCCCTGGGCAAGGCTCAGCC
dog (1148) --------------GAATCT------------TCTTGGCAATCCCTTAGGCCAGGCTCAGTT
bovine (1343) --------------GAATCC------------TCCTGGTAACCCCATGGGAAAAGTTCAGTT
pig (1183) --------------GAATCT------------GCCTGGTAACCCCGTGGG-AACGTTCAGTT
mouse (1502) ACAGTAACTGTTAGAATCCCAGCTGCAACTTCATGCCAGGTGCCAGGTCCATGCTCATCA
O
P
1801 1860
human (1374) CACAGGAGACAGAGGTCCAGAGAGG--CCTAGTGACTGGCCTGGGG-TCACACAGCA-GG
cat (1247) CACAGGAGACAGAGGCTCAGAGAGG--CTGAGCAACCGGCTTGGGGGTCACACAGCA-GG
dog (1184) CACAGGCGACAGAGGCTCAGAGAGG--CTGAGCACCTGGCTTGGGG-TCACACAGCAAGG
bovine (1379) CACAAAAGACAGAGGCTAAGAAAGA--CTGAGCAGTTGACCTAGGG-TCACACAGCC-CA
pig (1218) CACAGAAGACAGAGGCTCAGAGAGG--CTGAGCAACTGGCCTGGGG-TCACACAGCT-GG
mouse (1562) GTCAGG-GACTGGAACTCAGAGATCTCCTGGGAAA--G-CTTCAGTCTCACAGATTC---
Q
1861 1920
human (1430) GAAATGGCATAGGTGGGATTTGAACCTGCCTCTGACGAC----CCAGAGGCT-----GAG
cat (1304) GAAGTGGCACAGGTGGCATTTGAACCCAGCTCTGACGACT---CCCGCAGCT-----GAG
dog (1241) GAAATGGTAGAGGCAGGATTTGAACCCAGCTCTGCTGACT---TCAGCTGCT-----GGG
bovine (1435) GAGATGGCACGGATGAGATTTGAACCCAGCTCTGTTGACC---TCAGAGGCT-----GAG
pig (1274) GAACTGGCAGAGGTGGGATTTGAATCCAGCTCTGTTGACC---TCAGAGACT-----AAA
mouse (1615) -AAAAGCCAGAGA---GATCTAGTCACAGC-CTGGGGCCCAGAGCAGTGACTTAGGAGAG
R
S
1921 1980
human (1481) CTTTTC-TTCCCTTCTGAGCCCTGTGGGAGAACTGTCAGCAGGCAGAGGTGG--GGGCAG
cat (1356) CTCTTC-T---CAACTGAGCCCTGAGGGAGGTTGCCAGCAGACAGGGGTGG--GGACGG
dog (1293) CTTTTC-TTCTCAACTGAGCCCTGCAGGAGAGTGGTCAGCAAACAGAGGTGG--GGACGG
bovine (1487) CTGTTC-TT---AACTGTTCCTTGCAGGACAGGTGTCAGCACACAGAGGTGG--GGGCGG
pig (1326) TTGTTC-TTTTCAACTGTGCTCTGTGGGAAACGAGTTAGCAAACAG--GTGG--GGACGG
mouse (1670) CCGTGCCTTTTAAAGTGGACCTTGTAG---A--------CAGCCAGAGGTGGAGGGACTG
MR-1
1981 2040
human (1538) CTA-------CCCAGCCCTCC---CC-CGACCCAGCCCTTTGTCTTTGGATTGA---TAA
cat (1410) CCA-------CCCAGCCTTCC---CTTCCACCCTGCCCCTCATCCCTTGA---------A
dog (1350) CCA-------CGCAGCCTTCC---CTTCCACCCTCCCCCTCATCCCTTGACCGA---GCA
bovine (1541) GCA-------CTCAGCCCTCC---CTCCCACCCTGCCCTCCTCCCTCGACTGA---GCA
pig (1381) GCA-------CCCAGCCCTCC---TGCTCACCCTGCCAGTCATCCTTGGACCCA---GAA
mouse (1719) GGAGAAGTGGCTGAAGCCTCCAGACTCATTCCCACGCCCACATC--TGGACTAATTTGGA
