# Supplementary figures and images for: Differentiation and fiber type-specific activity of a muscle creatine kinase intronic enhancer
Source: Skelet Muscle. 2011 Jul 7;1:25. doi: 10.1186/2044-5040-1-25 (PMC3157005; doi:10.1186/2044-5040-1-25)

## Slide 1
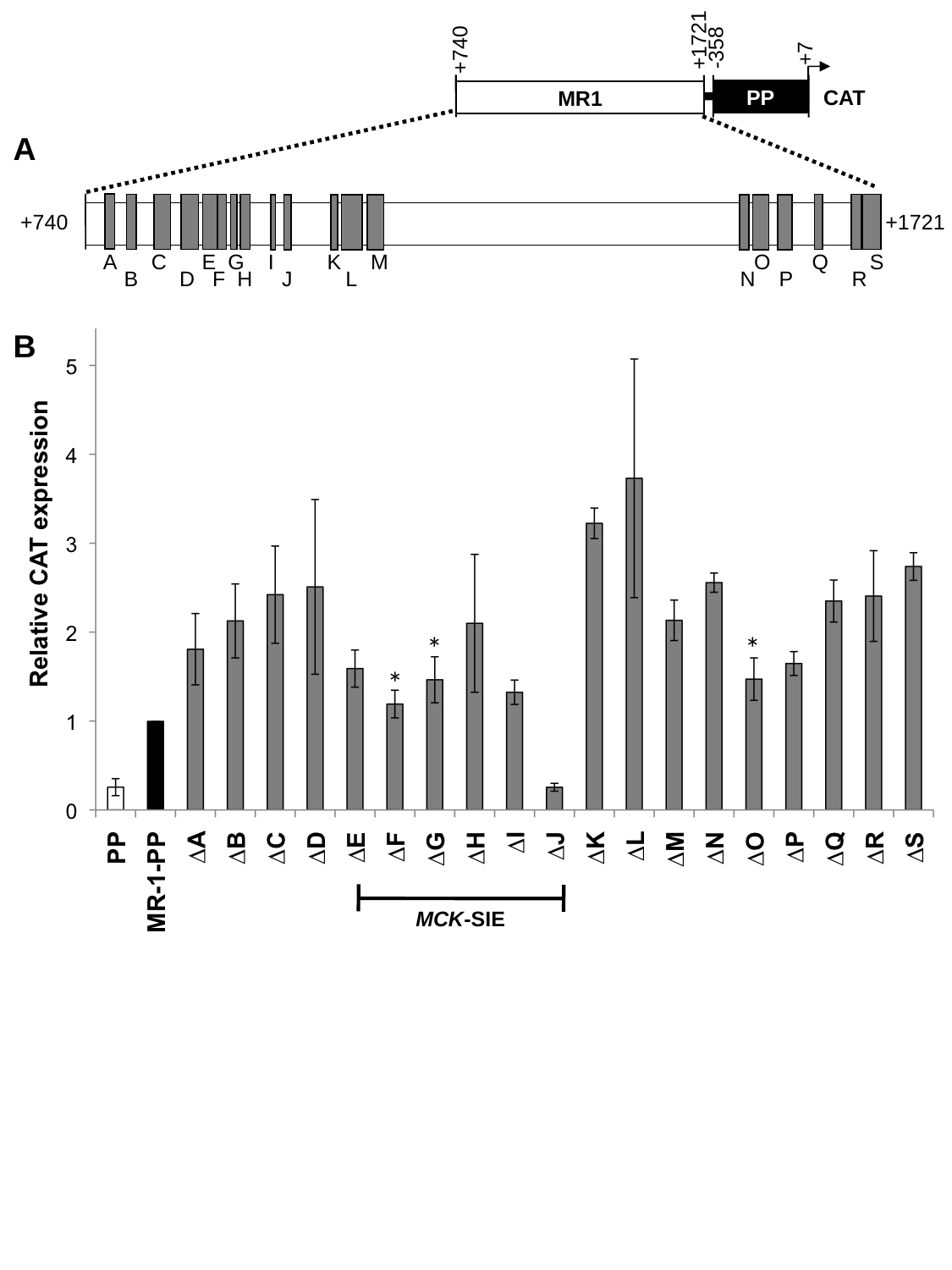

+1721
-358
+740
+7
CAT
PP
MR1
A
+740
+1721
A C E G I K M O Q S
B D F H J L N P R
B
*
*
*
MCK-SIE

Supplement: Additional file 2 — Figure S2. The functional consequence of individual deletions of the conserved 19 subregions throughout MR1. (A) Conserved regions within MR1 (gray blocks in part A, gray bars in part B) were deleted from MR1-proximal promoter-chloramphenicol acetyl transferase (MR1-PP-CAT) and tested for transcriptional activity in skeletal myocytes (gray bars). (B) MM14 cells were transiently transfected with constructs containing each of the 19 different conserved motif deletions, and cells were harvested as described in the Figure 2 legend. Relative CAT activity was normalized with the MCK 5'-enhancer alkaline phosphatase (AP) reference plasmid and compared to the intact MR1-PP-CAT (black bar) and to the PP-CAT (white bar). Expression levels of MR1-PP-CAT were scaled to equal 1.0. Asterisks indicate constructs that did not result in a statistically significant change in transcriptional activity. [file 2044-5040-1-25-S2.PPT]
